# Should artificial intelligence be used in conjunction with Neuroimaging in the diagnosis of Alzheimer’s disease?

**DOI:** 10.3389/fnagi.2023.1094233

**Published:** 2023-04-18

**Authors:** Sophia Mirkin, Benedict C. Albensi

**Affiliations:** ^1^Dr. Kiran C. Patel College of Osteopathic Medicine, Nova Southeastern University, Fort Lauderdale, FL, United States; ^2^Barry and Judy Silverman College of Pharmacy, Nova Southeastern University, Fort Lauderdale, FL, United States; ^3^St. Boniface Hospital Research, Winnipeg, MB, Canada; ^4^University of Manitoba, Winnipeg, MB, Canada

**Keywords:** Alzheimer’s disease, dementia, artificial intelligence, neuroimaging, diagnosis

## Abstract

Alzheimer’s disease (AD) is a progressive, neurodegenerative disorder that affects memory, thinking, behavior, and other cognitive functions. Although there is no cure, detecting AD early is important for the development of a therapeutic plan and a care plan that may preserve cognitive function and prevent irreversible damage. Neuroimaging, such as magnetic resonance imaging (MRI), computed tomography (CT), and positron emission tomography (PET), has served as a critical tool in establishing diagnostic indicators of AD during the preclinical stage. However, as neuroimaging technology quickly advances, there is a challenge in analyzing and interpreting vast amounts of brain imaging data. Given these limitations, there is great interest in using artificial Intelligence (AI) to assist in this process. AI introduces limitless possibilities in the future diagnosis of AD, yet there is still resistance from the healthcare community to incorporate AI in the clinical setting. The goal of this review is to answer the question of whether AI should be used in conjunction with neuroimaging in the diagnosis of AD. To answer the question, the possible benefits and disadvantages of AI are discussed. The main advantages of AI are its potential to improve diagnostic accuracy, improve the efficiency in analyzing radiographic data, reduce physician burnout, and advance precision medicine. The disadvantages include generalization and data shortage, lack of *in vivo* gold standard, skepticism in the medical community, potential for physician bias, and concerns over patient information, privacy, and safety. Although the challenges present fundamental concerns and must be addressed when the time comes, it would be unethical not to use AI if it can improve patient health and outcome.

## Introduction

Dementia, or Major Neurocognitive Disorder (MND), is a general term for any disease that causes a significant decline in at least one cognitive domain including memory, learning, executive function and additionally, impairs an individual’s ability to perform daily tasks (Emmady et al., 2022). Alzheimer’s disease (AD) is the most common form of dementia affecting an estimated 6.5 million Americans aged 65 years or older (Anonymous, 2022). With advancements in medicine, the size of the older U.S. population is continuing to grow and so too will the number of people living with the disease. By 2050, it is estimated AD will affect 12.7 million Americans over the age of 65 (Anonymous, 2022). The symptoms advance and worsen gradually, with changes to the brain occurring years before signs appear and often before receiving a clinical diagnosis. Dementia and its various forms can be classified and characterized in several ways. AD can be divided into three stages: early-stage (mild), middle-stage (moderate), and late-stage (severe; Alzheimer’s Association, 2022). Each phase serves to categorize the development of mental decline. In the mild stage, the person may begin to experience memory loss, poor judgment, the repetition of questions, misplacing items, and difficulty planning or organizing (Alzheimer’s Association, 2022). Signs of moderate stage disease involve increased memory loss and confusion, difficulty with language, difficulty organizing thoughts, hallucinations, paranoia, problems recognizing loved ones, tendency to wander, and mood changes such as anxiety and aggression (Alzheimer’s Association, 2022). As the disease progresses to the severe stage, the patient will be completely dependent on others for care and will display symptoms such as an inability to communicate, loss of awareness of their surroundings, weight loss, increased propensity to infections like pneumonia, and changes in physical abilities including walking, sitting, bowel control, and eventually swallowing (Alzheimer’s Association, 2022). Each person will experience different symptoms of the disease and may progress through the stages at a variable rate (Anonymous, 2018). The progression pace is influenced by age, genetics, biological sex, and other factors (Anonymous, 2022).

There is currently no cure for AD, leaving healthcare professionals to focus on slowing the progression of the disease to improve the quality of life of the patient. Although there is no cure, detecting AD in a timely and accurate manner is important because it allows for the development of an earlier treatment plan and care plan that may preserve cognitive function and prevent irreversible damage through intervention and lifestyle modifications (Crous-Bou et al., 2017). While a diagnosis can be made for AD based on clinical symptoms, there is currently no clinical standard to diagnose AD in the living that professionals agree on. A definitive diagnosis can be made postmortem with the identification of neurofibrillary tangles (NFT) or diffuse amyloid depositions known to be closely linked to the disease (McKhann et al., 2011). Improvements in blood biomarkers are also promising, but still underutilized. It is widely believed that the onset of neuropathological hallmarks of AD, such as NFT and abnormal amyloid plaques, begin to form years prior to the appearance of clinical symptoms (Guzmán-Vélez et al., 2022). Therefore, research has focused on the development of biomarkers and imaging to detect early signs in those most at risk. Examples include beta-amyloid and tau levels in the cerebrospinal fluid (CSF) and brain volume changes detectable by imaging (Counts et al., 2017). Neuroimaging has served as a critical tool in establishing diagnostic indicators of AD during the preclinical stage allowing for earlier diagnosis and intervention (Márquez and Yassa, 2019). The diagnostic imaging modalities most widely used in the diagnosis of neurodegenerative diseases are magnetic resonance imaging (MRI), computed tomography (CT) and positron emission tomography (PET; Schwarz, 2021). However, as neuroimaging technology quickly advances, there is a challenge in analyzing and interpretating vast amounts of brain imaging data (Carrillo et al., 2013). Given these limitations, there is great interest in using computer-aided algorithms for integrative analysis, namely artificial intelligence.

Artificial intelligence (AI) is a field of developing computer programs that simulate human functioning. There are two subsets that have been used significantly in AD research - machine learning (ML) and deep learning (DL). Illustrating a simple definition, machine learning uses algorithms to recognize patterns from data and applies that knowledge to reach solutions and make predictions for new information (Silva-Spínola et al., 2022). The commonly used learning processes are supervised and unsupervised learning. Supervised learning trains the algorithm using labeled input data with known output data until the model can correctly detect underlying patterns between the datasets (Choi et al., 2020). The trained model is then presented with data it has never seen before, known as test data, to assess how accurately the algorithm can make future predictions on unlabeled data. In contrast, unsupervised learning trains an algorithm using unlabeled data where the correct output variable is unknown. Here, the algorithm freely determines whether patterns exist within the dataset without human intervention (Choi et al., 2020). Deep learning, a more complex subset of ML, uses a convolutional neuronal network architecture to analyze data in a logical form similar to how the human brain functions (Zaharchuk et al., 2018). Neural networks include nodes that work as neurons that can recognize and classify patterns from data while continuously learning and improving over time (Choi et al., 2020). Deep learning differs from machine learning mainly by the use of neural networks, a low need for human intervention, and larger data requirements. Most importantly, while raw data is usually preprocessed before applying ML, DL can process raw input data directly. There are various artificial intelligence techniques in Alzheimer’s disease detection that are outlined by Subasi (2020). For example, ensemble classifier, support vector machines (SVMs), and Random Forest are one of many techniques used in recent studies. Research has focused on using ML and DL technology to create algorithms that recognize, process, and extract data from neuroimaging to detect AD with a high specificity and sensitivity (Silva-Spínola et al., 2022). For instance, Kapadnis et al. (2023) discusses the use of deep feature extraction methods for early AD detection. AI introduces limitless possibilities in the future diagnosis of AD, yet there is still resistance from the healthcare community to incorporate AI in the clinical setting. This is due to fears such as AI at some point displacing certain physicians like radiologists (Ahuja, 2019). The goal of this paper is to answer the question of whether AI should be used in conjunction with neuroimaging in the diagnosis of AD. To answer this question, we will examine the current modalities used to detect AD and their limitations, current AI research in AD, and the pros and challenges of AI.

## Current diagnostic imaging and Its limitations

### Magnetic resonance imaging

Magnetic resonance imaging (MRI) uses powerful magnets to align protons along a magnetic field (Magnetic Resonance Imaging (MRI), 2022). A radiofrequency current stimulates and spins the protons out of equilibrium in the patient. When the current is turned off, the protons realign with the magnetic field and release energy detected by the MRI sensors. The image produced depends on the time it takes for the protons to realign with the field and the energy released (Magnetic Resonance Imaging (MRI), 2022). MRI has been largely used in the clinical identification of AD due to its ability to provide detailed information about brain structure *in vivo*.

The diagnostic guidelines of AD created by the National Institute on Aging and the Alzheimer’s Association recommend the use of structural MRI (sMRI) in its criteria, highlighting its integral role in the clinical assessment of patients with suspected AD (Frisoni et al., 2010; Albert et al., 2011). sMRI assesses brain atrophy and tissue changes with its capability to differentiate between grey matter and white matter (Ferreira and Busatto, 2011). Structural MRI studies of patients with AD have revealed atrophy in medial temporal lobe structures including the hippocampus, amygdala, entorhinal cortex (ERC) and parahippocampal gyrus (Ferreira and Busatto, 2011). Medial temporal lobe atrophy (MTA) is associated with lower executive function, general cognitive function, and episodic memory performance (Oosterman et al., 2012). Studies have found ventricular enlargement, whole brain atrophy and cortical thickness reduction in patients with AD (Lerch et al., 2005; Nestor et al., 2008; Evans et al., 2010). WMH were initially thought to be associated with small vessel cerebrovascular disease. However, recent literature suggests there to be an association with neurodegeneration in AD. T2-weighted MRI and Fluid-Attenuated Inversion Recovery (FLAIR) MRI have been used to detect white matter hyperintensities (WMH) and have highlighted larger volume changes in the periventricular and posterior regions in patients with AD (Damulina et al., 2019; Garnier-Crussard et al., 2022). Although the relation between white matter lesions and amyloid accumulation is unclear, a study suggests WMH may potentially help identify early amyloid accumulation in patients (Moscoso et al., 2020). Despite its usefulness, there are several limitations to structural MRI. First, although brain atrophy is correlated with both tau deposition and neuropsychological symptoms, structural MRI cannot directly observe the effect of amyloid plaques or NFTs in the brain (van Oostveen and de Lange, 2021). Second, loss of hippocampal volume is not AD specific and is found in Parkinson’s disease (Camicioli et al., 2003), schizophrenia (Koolschijn et al., 2010), traumatic brain injury (Bigler et al., 1997), temporal lobe epilepsy (Duan et al., 2020), and depression (Shamim et al., 2009). Third, although there are characteristic patterns of brain loss in AD, cerebral atrophy is a nonspecific result of neuronal damage (Johnson et al., 2012). Fourth, an atypical form of AD spares hippocampal atrophy; therefore, sMRI may not be useful in detecting atypical forms of the disease in the early stages (Ferreira et al., 2017; Firth et al., 2019). Lastly, MRI may not be tolerated by claustrophobic patients and a CT scan may be needed instead. Although, open MRI systems and larger bore sizes may help reduce claustrophobia in some individuals (Sammet, 2016).

Other more advanced MR techniques that are not used in routine clinical settings but serve an important role in AD research include functional magnetic resonance imaging (fMRI) and diffusion tensor imaging (DTI). fMRI is based on blood-oxygen level dependent (BOLD) changes in the brain that occur during specific tasks (Orringer et al., 2013) and has been widely used to study pathophysiologic changes seen in memory loss in AD. A meta-analysis of fMRI activation during episodic memory in AD and MCI showed hypoactivation of the medial temporal lobe structures in AD and hyperactivation in MCI (Terry et al., 2015). Hyperactivation in MCI is thought to reflect inefficient compensatory activity early in the disease (Dickerson and Sperling, 2008). Although fMRI provides insight into pathophysiology, it is not recommended in the clinical setting due to its sensitivity to head motion (Hausman et al., 2022) which is problematic in older adults, low signal or contrast to noise ratio (Chandra et al., 2019), and lack of validity of BOLD signal as a measure of neuronal activity (Specht, 2020). DTI is used to assess the microstructural integrity of cerebral WM fiber tracts based on water diffusion within the brain. Fractional anisotropy (FA) and mean diffusivity (MD) are metrics that measure the directionality of water diffusion and the mean water diffusion rate, respectively (Okudzhava et al., 2022). A meta-analysis of DTI in MCI and AD individuals reported MD was increased in AD in all regions tested (frontal lobe WM, temporal lobe WM, parietal lobe WM, occipital lobe WM, hippocampus, cingulum bindle and cingulate cortex WM, corpus callosum, superior longitudinal fasciculus, uncinate fasciculus, and the posterior limb of the internal capsule) and FA was decreased in all regions except parietal white matter and internal capsule (Sexton et al., 2011). Limitations in DTI include the need for more large cohort studies to validate the findings, low signal to noise ratio (Ranzenberger and Snyder, 2022), and variability of DTI-based diffusion metrics between MRI scans which imposes a major restriction in multicenter studies (Sheikh-Bahaei et al., 2017).

### Computed tomography

Computed tomography is a computerized x-ray imaging procedure that generates cross-sectional images or “slices” (Computed Tomography (CT), 2022). A narrow beam of x-rays is aimed at a patient and quickly rotated around the body to produce signals that are processed by the machine’s computer. Several slices can be taken consecutively and are then digitally stacked together to form a three-dimensional image of the patient (Computed Tomography (CT), 2022). CT is not recommended for first-line imaging as it is less sensitive in detecting changes associated with cognitive impairment compared to MRI. However, there are still many advantages such as lower cost, shorter acquisition time, and wider availability (Pasi et al., 2011; Health Quality Ontario, 2014).

Under current recommendation and guidelines, structural imaging (MRI or CT) is required for evaluation of patients presenting with cognitive symptoms in the clinical setting (Sheikh-Bahaei et al., 2017). CT reveals the anatomic structure of the brain to detect brain atrophy and rule out other abnormalities that can be mistaken as AD such as tumors, hydrocephalus, and chronic subdural hematoma (Frisoni, 2001). Serial CT imaging has been used to track and observe changes as the disease progresses. A CT-based longitudinal study on veterans tracked the progression of AD over 4–6 years. Absolute brain volume loss accelerated 1.5x faster for AD patients versus non-AD patients (Bin Zahid et al., 2016). The study highlights the possibility of using CT to monitor the progression of cognitive decline and dementia however, more recent studies have found modalities such as MRI or PET to serve a more vital role in tracking disease progression (van Oostveen and de Lange, 2021). A CT study detected enlargement of the third and lateral ventricles in AD patients and found a correlation between the rate of neuropsychological decline and the rate of ventricular enlargement (Luxenberg et al., 1987). A recent study tracked the growth of ventricular cerebrospinal fluid (vCSF) to assess if ventricular expansion can serve as a reliable indicator of neurodegeneration (Adamo et al., 2020). Furthermore, one field of medicine that has sparked interest in researchers to potentially treat Alzheimer’s is low dose ionizing radiation such as with the use of CT scans (Jebelli et al., 2022). For example, a case study presented a patient with early-stage AD that was treated with low doses of ionizing radiation to the brain. After receiving four CT scans, the patient’s mental clarity improved and even exhibited restored function of clarinet jazz-playing. The treatment was discontinued due to fears of adverse events however, further research is needed to assess if CT scans could serve a role in temporarily improving the quality of life of patients with AD in the future (Cuttler et al., 2022; Jebelli et al., 2022). There are various limitations to CT scans when compared to MRI. MRI is more sensitive at detecting focal atrophic changes in the nuclei (Sheikh and Glick, 2022), more sensitive to white matter changes (Johnson et al., 1987) and does not require ionizing radiation (Pearce et al., 2012). In addition, comparative analysis suggests MRI may be more accurate than CT for distinguishing AD from other conditions such as vascular dementia although the study is not conclusive (Beynon et al., 2012). MRI remains the preferred first-line modality; however, both play a key role in ruling out structural lesions of the brain in individuals with dementia (Alzheimer's Association et al., 2003; Brisson et al., 2020).

### Positron emission tomography

Positron emission tomography (PET) scans work at the molecular level to produce three-dimensional images that depict biochemical and molecular processes (Herring, 2011). A PET radiotracer attaches to the molecular target which then allows for the measurement of various processes such as metabolism, blood flow, and regional chemical composition in the body (Kapoor and Kasi, 2022). PET radiotracers have been developed in the field of AD to meet the increasing need for early detection and treatment monitoring of the disease. The following section will provide a brief overview of current PET scans available for AD imaging, namely FDG-PET, amyloid-PET, and tau-PET (Bao et al., 2021).

Fluorodeoxyglucose, an analog of glucose, is introduced to the patient intravenously to measure brain metabolism. Glucose is the principal source of energy for the human brain. Any changes to neural activity in neurodegeneration will be reflected by glucose consumption (Minoshima et al., 2022). The term FDG uptake refers to the amount of radiotracer uptake. Areas of low radiotracer uptake are associated with lower brain activity and produce darker spots (hypometabolism) on images. The standardized uptake value (SUV) is a commonly used method to assess the degree of FDG uptake in a region of interest in PET imaging. SUV is calculated as the ratio of tissue activity concentration and administration dose, divided by body weight (Nugent et al., 2020). The ratio of SUV data from two different regions within the same PET image is referred to as the SUV ratio (SUVr). SUVr also serve an important role in quantifying tracer uptake in amyloid and tau imaging (Chiao et al., 2019). The characteristic manifestation of AD on FDG-PET is hypometabolism in the posterior cingulate cortex (PCC), precuneus (PrC), parietotemporal cortex, and in the frontal cortex in advanced stages (Minoshima et al., 1997; Herholz et al., 2002; Bao et al., 2021). FDG-PET has provided clinical value in detecting distinct patterns of cortical hypometabolism in AD (Dave et al., 2020), differentiating between other neurological diseases (Shivamurthy et al., 2015), and in predicting MCI conversion to AD (Smailagic et al., 2018). Additionally, the modality has been used to identify subtypes of AD based on hypometabolic regions including a “typical” subtype, “limbic-predominant” subtype, and a rare “cortical-predominant” subtype exhibited in younger individuals with more severe executive impairments (Levin et al., 2021). Statistical parametric mapping (SPM) is a statistical technique used for evaluating brain activity during functional neuroimaging studies such as fMRI and PET. SPM allows for comparison of SUV in a region of interest of a patient to a normal cohort (Smith et al., 2022). Additionally, it has been shown SPM for FDG-PET can increase diagnostic performance in AD (Ford et al., 2021). FDG-PET is widely available, has a relatively low cost (Minoshima et al., 2022) and serves a vital role in AD research and diagnosis. Despite its usefulness, limitations include its requirement of intravenous access and patient exposure to radioactivity (Johnson et al., 2012). Furthermore, hypometabolism is a sign of neurodegeneration and therefore it may not be useful for detecting early stages of AD before neuronal damage has occurred (Drzezga et al., 2018).

Amyloid-PET enables *in vivo* detection of amyloid deposits in the brain, one of the neuropathological hallmarks of AD. Currently, three amyloid PET tracers are approved by the FDA for clinical use: ^18^F-Florbetaben (Neuraceq), ^18^F-Florbetapir (Amyvid), and ^18^F-Flutemetamol (Vizamyl; Anand and Sabbagh, 2017). Amyloid accumulation is commonly assessed with SUVr quantification though this technique has been shown to overestimate amyloid burden in cognitively normal individuals (Golla et al., 2019; Krishnadas et al., 2021). The modality has played a major role in diagnosing AD, differentiating AD from other neurodegenerative conditions, and predicting the risk of progression of MCI to AD dementia (Krishnadas et al., 2021). A VA-led research study found amyloid PET scans to be useful in ruling out AD in individuals without amyloid buildup and served an important role in the clinical care and management of older veterans with AD (Turk et al., 2022). Although AD is known to be a disease of grey matter pathology, white matter abnormalities play an important role in the pathological changes seen in AD (Pietroboni et al., 2022). Various studies have used amyloid PET tracers to assess myelin changes in cerebral white matter (Moscoso et al., 2022; Pietroboni et al., 2022). A major limitation is the fact that a positive amyloid-PET scan is not sufficient to diagnose AD. It serves merely as a specific and sensitive tool that can assess the likelihood of a diagnosis (Kolanko et al., 2020). Furthermore, a study concluded that FDG-PET was better at assessing the progression and severity of MCI and Alzheimer’s compared to florbetapir-PET scans (Khosravi et al., 2019). The study supports the idea that amyloid plaque deposition and cognitive impairment are poorly correlated in AD (Bucci et al., 2021). Therefore, amyloid imaging may serve a limited role in the future for assessing cognitive decline in patients. The need for a more reliable marker has shifted research towards another pathology that may better help diagnose and monitor AD progression- tau tangles.

Tau is a protein that accumulates in the brain of individuals with AD and other forms of dementias. Recently, the FDA has approved Tauvid (flortaucipir F18) for PET imaging of the brain to assess the distribution of aggregated tau neurofibrillary tables (NFT), another neuropathological hallmark of AD (Jie et al., 2021). The distribution of tau proteins deposits has been shown to be more closely associated to cognitive decline when compared to amyloid (Hanseeuw et al., 2019). While amyloid tracers tend to have a wide distribution in the neocortex, there tends to be higher levels of tau radiotracer retention in the inferior lateral temporal and parietal cortices of AD patients (Yousefzadeh-Nowshahr et al., 2022). Tau PET has also been used to differentiate AD dementia from other neurodegenerative diseases such as frontotemporal lobar degeneration (FTLD) disorders based on the location of tau protein in the brain (Abbasi, 2020). A study found cognitively unimpaired individuals positive for tau and amyloid were at high risk for cognitive decline in the short term, with tau burden in the MTL and neocortex region displaying a substantial additional risk (Ossenkoppele et al., 2022). Despite its utility, limitations of tau imaging include reports of *in vivo* off-target binding (Smith et al., 2022), variability of thresholds for tau positivity rates between studies (Weigand et al., 2022), and similarly as with amyloid, a positive tau marker alone is not sufficient for an AD diagnosis.

## Current use of AI in AD research

Brain imaging modalities like MRI, CT, PET, as well as molecular biomarkers, such as amyloid plaques and tau in CSF, are used in clinical settings to identify a patient’s cognitive status (Gunes et al., 2022). However, as noted, limitations exist when using neuroimaging alone to identify AD. With advancing technology, there is a challenge in analyzing and interpretating vast amounts of brain imaging data. The use of AI with neuroimaging for the diagnosis of AD is a rapidly emerging field and has the potential to solve these problems. AI has the ability to integrate complex multimodal data, improve the accuracy of biomarker-based testing, and has a promising future of providing accurate and widely accessible early diagnosis of AD (Gunes et al., 2022). Below is a review of studies (see Table 1) that have developed AI-based algorithms for classifying, monitoring, and diagnosing AD as well as studies that have identified non-invasive, early AD biomarkers.

**Table 1:** Summary of artificial intelligence research in Alzheimer’s disease.

Name of article	Year	Modality	AI	Objective	Result
Multimodal and Multiscale Deep Neural Networks for the Early Diagnosis of Alzheimer’s Disease using Structural MR and FDG-PET Images	2018	MRI and FDG-PET	DL	Propose a new deep-learning-based framework to identify individuals with AD using a multimodal and multiscale deep neural network	84.4% accuracy in identifying individuals with MCI who will convert to AD at 3 years before conversion. 94.23% sensitivity in classifying individuals with clinical diagnosis of probable AD. 86.3% specificity in classifying non-demented controls.
Earlier Detection of Alzheimer Disease using N-fold Cross Validation Approach	2018	MRI	ML	The early detection of Alzheimer Disease detection system using N-Fold Cross Validation Approach is analyzed	The N-fold cross validation method successfully recognized AD disease in brain MRI images from ADNI with 99.26% accuracy and minimum error rate.
Machine Learning Based Hierarchical Classification of Frontotemporal Dementia and Alzheimer’s Disease	2019	MRI	ML	Develop a machine learning-based automated classifier for differential diagnosis of CN, AD, and FTD subtypes	75.8% classification accuracy of the entire hierarchical classification tree. Classifier was successful in discriminating among CN, AD, and FTD subtypes. 90.8% accuracy in discriminating FTD from AD.
A Deep Learning Model to Predict a Diagnosis of Alzheimer Disease by using (18)F-FDG PET of the Brain	2019	FDG-PET	DL	Develop and validate a deep learning algorithm that predicts MCI, final diagnosis of AD, or neither	Achieved 82% specificity at 100% sensitivity in predicting a AD diagnosis in an average 75.8 months prior to the final diagnosis
Ultra-Low-Dose (18)F-Florbetaben Amyloid PET Imaging Using Deep Learning with Multi-Contrast MRI Inputs	2019	Amyloid-PET/MRI	DL	Reduce radiotracer requirements for amyloid PET/MRI without worsening diagnostic quality	Using deep learning methods, simultaneously acquired MR images and ultralow-dose PET data produced high-quality amyloid PET images
Automatic Detection of Alzheimer Disease Based on Histogram and Random Forest	2019	MRI	ML	Obtaining a reliable and fast model for automatic AD detection	Using the histogram as a feature extractor and Random Forest as the classifier, the model yielded an accuracy rate of 85.77% in identifying AD.
Predicting Sporadic Alzheimer’s Disease Progression *via* Inherited Alzheimer’s Disease-Informed Machine Learning	2020	sMRI, amyloid-PET, FDG-PET	ML	Develop cross-validated multi-biomarker model to predict the rate of cognitive decline in AD	Multi-biomarker model predicted 4-year rate of decline in global cognition and memory in sporadic AD
Linguistic Markers Predict Onset of Alzheimer’s Disease	2020	N/A	ML	Study linguistic performance as an early biomarker of AD in CN subjects	Future onset of AD was associated with telegraphic speech, repetitiveness, and misspellings. Study demonstrates it is possible to predict future onset of AD using language samples obtained from CN individuals.
A Machine Learning Approach for the Differential Diagnosis of Alzheimer and Vascular Dementia Fed by MRI Selected Features	2020	fMRI and DTI	ML	To develop an algorithm to classify vascular dementia and AD in patients with a “mixed VD-AD dementia” clinical profile	Adaptive neuro-fuzzy inference system (ML algorithm) achieved an 84% classification accuracy and a correct prediction rate of 77.33%. The study demonstrates high discriminative ability in classifying AD and VD profiles.
A 3D Densely Connected Convolution Neural Network with Connection-Wise Attention Mechanism for Alzheimer’s Disease Classification	2021	MRI	DL	Developed a densely connected convolution neural network to discriminate AD versus healthy subjects, MCI converters versus healthy subjects, and MCI converters versus non-converters	97.35% accuracy in discriminating AD patients from healthy controls. 87.82% accuracy in discriminating MCI converters against healthy controls. 78.79% accuracy in distinguishing MCI converters against non-converters.
Analysis of Features of Alzheimer’s Disease: Detection of Early Stage from Functional Brain Changes in Magnetic Resonance Images using a Finetuned ResNet18 Network	2021	fMRI	DL	Proposes a deep learning-based method that can predict MCI, early MCI, late MCI, and AD	ResNet18 network achieved 99.99% accuracy on EMCI vs. AD, 99.95% accuracy on LMCI vs. AD, and 99.95% accuracy on MCI vs. EMCI classification.
Association of Digital Clock Drawing with PET Amyloid and Tau Pathology in Normal Older Adults	2021	PET	ML	Determine whether a digital clock-drawing test can help discriminate diagnostic groups and detect amyloid and tau pathology in CN older adults	DCTclock was able to differentiate CN from MCI and early AD. Additionally, the study provides Class II evidence that DCTclock results were associated with amyloid and tau pathology in CN older adults.
Performance of Machine Learning Algorithms for Predicting Progression to Dementia in Memory Clinic Patients	2021	N/A	ML	Test if ML algorithms accurately predict 2-year dementia incidence in memory clinic patients	Using only 6 variables, ML algorithms reached 90% accuracy in predicting incident dementia within 2 years compared with 2 existing predictive models.
Early-Stage Alzheimer’s Disease Prediction Using Machine Learning Models	2022	MRI	ML	Develop a model to distinguish true AD affected people from a given population	The best validation model in the study achieved an average accuracy of 83% on the test data of AD.
Classification of Alzheimer’s Disease Based on Abnormal Hippocampal Functional Connectivity and Machine Learning	2022	fMRI	ML	Study the functional connectivity (FC) of the hippocampus and other brain structures to distinguish MCI, AD, and CN subjects	AD and MCI subjects demonstrated reduced FCs between the hippocampus and left insula, left thalamus, cerebellum, right lingual gyrus, posterior cingulate cortex, and precuneus. ML model achieved discriminative performance of 82.02% accuracy in AD vs. NC, 81.33% accuracy in MCI vs. NC, and 81.08% in AD vs. MCI
Machine Learning Based Multimodal Neuroimaging Genomics Dementia Score for Predicting Future Conversion to Alzheimer’s Disease	2022	MRI	ML	Develop and analyze biomarkers that can help predict the progression and development of AD	Genetic data could better predict future AD progression for CN subjects. MRI can better characterize subjects with stable MCI
A Predictive Model Using the Mesoscopic Architecture of the Living Brain to Detect Alzheimer’s Disease	2022	MRI	ML	Propose a method to characterize early and later forms of AD	The model reliably discriminates between people with (ADrp) and without (nADrp) AD related pathologies with 98% accuracy.
ArtifactID: Identifying Artifacts in Low-Field MRI of the Brain Using Deep Learning	2022	MRI	DL	Develop a model to identify wrap-around and Gibbs ringing in low-field brain MRI	ArtifactID model was able to identify and localize wrap-around. Achieved mean precision and recall metrics of 97.6 and 92.83%.
Alzheimer’s Disease Detection using Artificial Intelligence	2022	MRI	DL	Develop an AD detection approach using deep learning models	The results show that CNN achieved a testing accuracy of 95.70% and a validation accuracy of 99.71% for the diagnosis of AD from brain MRI scans

Machine learning has been used in the disease classification of AD. A study developed a ML algorithm to classify AD based on abnormal hippocampal functional connectivity (Zhu et al., 2022). One-hundred nineteen subjects aged 60–85 years were assessed with functional MRI and assigned to AD, MCI, or normal control groups. A SVR model yielded 82.02%, 81.33%, and 81.08% accuracy in discriminating AD vs. NC, MCI vs. NC, and AD vs. MCI, respectively, (Zhu et al., 2022). Another study developed a densely connected convolutional neural network with a connection-wise attenuation mechanism (CAM-CNN) to predict AD diagnosis with higher accuracy using MR brain scans (Zhang et al., 2021). The method achieved 97.35%, 87.82%, and 78.79% accuracy for distinguishing mild AD patients, MCI converters, and stable MCI subjects versus healthy controls (Zhang et al., 2021). An additional study used deep learning methods like CNN to develop a model that could detect AD from MRI scans, yielding a testing accuracy of 95.70% and a validation accuracy of 99.41% (Subasi et al., 2022). Studies have incorporated ML to differentiate AD from other diseases such as frontotemporal dementia (Kim et al., 2019) and vascular dementia (Castellazzi et al., 2020). Furthermore, a study created a multimodal deep neural network using structural MR and FDG-PET images for the early diagnosis of AD (Lu et al., 2018). The method delivered a 94.23% sensitivity in classifying individuals with clinical diagnosis of probable AD, 86.4% accuracy in identifying MCI individuals who will convert to AD within 1 to 3 years, and 86.3% in classifying non-demented controls (Lu et al., 2018). Another study aimed to use ML techniques to develop a simple and fast model for automatic AD detection. The researchers utilized histogram as the feature extractor and Random Forest as the classifier. The results yielded a high accuracy rate of 85.77% in discriminating AD subjects from the control subjects (Alickovic, 2020). Lastly, a deep learning-based method was developed to predict early MCI (EMCI), late MCI (LMCI) and AD using fMRI dataset consisting of 138 subjects (Odusami et al., 2021). The proposed model performed better than other known models achieving an accuracy of 99.99% in EMCI vs. AD, 99.95% in LMCI vs. AD, and 99.95% in MCI vs. EMCI (Odusami et al., 2021).

Machine learning can create models from a combination of AD biomarkers to make a more accurate diagnosis. One study developed and analyzed novel biomarkers to help predict the progression of dementia of Alzheimer’s type (DAT; Mirabnahrazam et al., 2022). Brain MRI and SNP-based genetic data of a total of 543 patients were used in the study. The results showed that the genetic data could better detect the DAT progression compared to MRI, while the MRI data reflected anatomical changes to the brain and was better able to categorize subjects with MCI impairment. By combining the genetic and MRI data, the model was better able to predict AD progression than using either method alone (Mirabnahrazam et al., 2022). Furthermore, previous studies have shown cognitive decline is associated with higher amyloid-PET (Koscik et al., 2020), hippocampal atrophy on MRI, hypometabolism on FDG-PET, and greater tau levels (Mielke et al., 2017). A study attempted to merge the multimodal data by developing a biomarker-based machine learning model to predict the progression of cognitive decline in AD (Franzmeier et al., 2020). First, a SVR model was trained using amyloid-PET, FDG-PET, sMRI, and CSF data from 121 autosomal dominant AD (ADAD) patients to predict the estimated years for symptoms to develop. ADAD patients were chosen as the training sample because these individuals tend to develop AD at an earlier age, thus age-related pathologies would not confound the findings of cognitive decline. After model training, the model was applied to a sample of 216 patients with sporadic AD patients to test if it could predict cognitive decline. The results show the model was able to predict with high accuracy the 4-year rate of global cognitive and memory decline in sporadic AD individuals (Franzmeier et al., 2020). Evidently, the capacity of ML algorithms to integrate multimodal data has been essential in AD research.

Not only has AI been used to classify and measure the progression of AD, but a recent study has combined neuroimaging with a deep learning algorithm to predict a diagnosis of AD in individuals presenting with first signs of memory impairment (Ding et al., 2019). FDG-PET brain images of 1,002 patients taken from 2005 to 2017 were collected from the Alzheimer’s Disease Neuroimaging Initiative (ADNI) database. The proposed method, a convolutional neural network of InceptionV3, was able to predict the final clinical diagnosis of AD in an average of 75.8 months prior to the final diagnosis with 82% specificity at 100% sensitivity. The study demonstrates how a deep learning algorithm can improve the accuracy of predicting the diagnosis of AD using brain FDG-PET and if used clinically, would provide a wider window for earlier therapeutic intervention (Ding et al., 2019). Another study utilized the N-fold cross validation approach to detect early signs of AD from a collection of MRI brain images. The approach was found to be most effective at classifying AD from brain MRI images with minimal error rate when compared to other traditional methods, yielding a 99.26% accuracy rate (Sampath and Indumathi, 2018). Lastly, a recent study developed a machine learning algorithm to predict early-stage AD using MRI data from 150 patients aged 60 to 96 (Kavitha et al., 2022). The patients were classified as either “non-demented” or “demented” and applied to five classifier ML models including decision tree (DT), random forest (RF), support vector machine (SVM), XGBoost, and Voting. Four performance metrics, including accuracy, precision, recall, and F1 score, were evaluated for each model. At the end of the study, the patients received their result indicating the current stage of AD he or she was currently in. According to the results, the study found men were more likely to have cognitive decline than females, demented patients were less educated, brain volumes were greater in non-demented patients, and a greater number of demented patients were in the 70 to 80 age group. The authors hope to use new metrics in the future to train the ML model to detect early AD with better accuracy (Kavitha et al., 2022).

Studies have also used machine learning to test non-invasive, early biomarkers of AD. One study aimed to use ML to predict the future onset of AD through automated linguistic analysis (Eyigoz et al., 2020). A study of 270 participants were asked to perform the cookie-theft picture-description task in which the participants were asked to write a description of the picture. All the participants were cognitively normal when performing the test and 50% later developed AD symptoms before age 85. The results show that the future onset of AD is associated with telegraphic speech, repetitiveness, misspellings, and lack of punctuation (Eyigoz et al., 2020). The study demonstrates that simple, inexpensive speech tests can be used as a future tool for early detection and monitoring the progression of AD. Additionally, a study tested whether a digital clock-drawing test, DCTclock, can detect AD biomarkers in individuals with no symptoms (Rentz et al., 2021). Three-hundred participants were asked to perform the DCTclock test, the 30-min pen-and-paper based cognitive tests (PACC) and underwent PET brain scans. The results showed that the DCTclock test outperformed the PACC and had a significant correlation with PET imaging scans at detecting evidence of amyloid plaques in asymptomatic individuals. With ML, the researchers could analyze not only if the participants were correctly drawing a clockface, but could also evaluate features of the drawing process, including their movements, to reveal subtle signs of cognitive impairment (Rentz et al., 2021). Lastly, an innovative technological company named Canary Speech is using AI and ML to build voice models to identify AD and other cognitive diseases in 20 to 30 s samples of speech (Canary Speech, 2022). The technology hopes to use language and speech biomarkers to provide an accurate assessment and diagnosis for individuals in the future.

AI with neuroimaging is a rapidly emerging field and continues to be extensively tested in AD research. However, these models are not currently available in routine clinical practice, but the progression towards use in clinical settings is expected as technology advances. In the next section, possible benefits and disadvantages of using AI in the clinical setting will be discussed.

## Pros of AI

### Improving diagnostic accuracy

**Figure 1:**
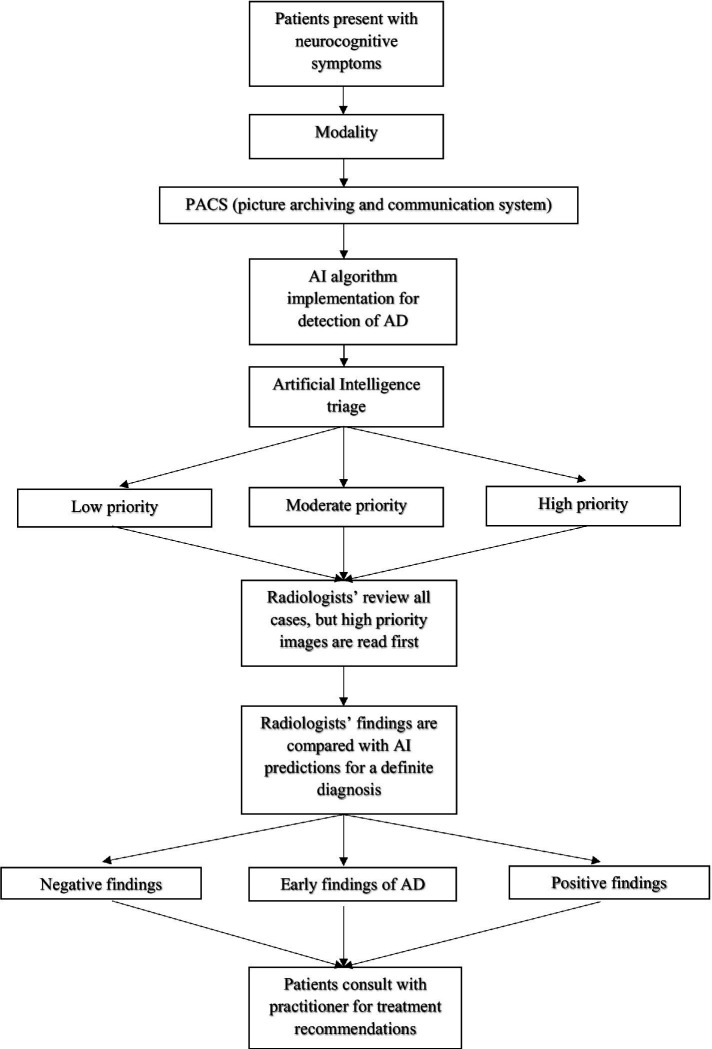
Legend: Patients present with neurocognitive symptoms ranging from noticeable memory loss, trouble performing routine tasks, confusion, personality changes, etc. A brain scan is performed in the clinic using the appropriate modality such as CT, MRI, or PET. The image is stored into the picture archiving and communication systems (PACS) database and processed into an artificial intelligence-based system to detect and sort low, moderate, and high priority brain scans. High priority cases are reviewed first by the radiologists followed by moderate and low cases. Once a diagnosis is made, the findings are compared with the AI database. If the radiologist’s decision does not match with the AI system, a second opinion is needed before making a definite diagnosis. Once a definite diagnosis is made, the patients can consult with their practitioner for future treatment recommendations.

AI has the potential of improving diagnostic accuracy in the clinical setting (Figure 1). A largescale study performed at the University of Exeter tested if machine learning algorithms could accurately predict the progression of dementia in 2 years (James et al., 2021). In a sample of 15,307 participants who attended a network of 30 National Alzheimer Coordinating Center memory clinics across the United States between 2005 and 2015, 1,568 received a diagnosis of dementia within 2 years of their initial assessment. The population was divided into 4 dementia subtypes: Alzheimer’s dementia (1,285 participants), Lewy Body dementia (82 participants), vascular dementia (21 participants), and other dementia subtypes (180 participants). The ML algorithm had an accuracy of 92% in predicting a 2-year incidence of dementia, presenting far more accuracy than other existing dementia risk prediction models such as Cardiovascular Risk Factors, Aging, and Incidence of Dementia (CAIDE) Risk Score and Brief Dementia Screening Indicator (BDSI). Additionally, the model identified 84% of individuals who were possibly misdiagnosed with dementia who then had their diagnosis reversed to a MCI or cognitively unimpaired diagnosis. Thus, not only can the ML algorithm accurately tell clinicians who will go on to develop dementia, but the algorithm could also help reduce the number of people who have been falsely diagnosed. The study demonstrates ML algorithms can be helpful in the decision-making process and be used as a potential diagnostic and validation tool in clinical settings (James et al., 2021). Furthermore, a team led by Dr. Marianna Inglese of the Imperial College created an algorithm based on T1-weighted MRI data of brain shrinkage to test the effectiveness of diagnosing AD in the early stages (Inglese et al., 2022). The current standard to diagnose AD involve lengthy brain scans to test for hippocampal atrophy and protein deposits as well as cognitive tests. The team used data taken from 783 individuals scanned with 1.5 tesla MRI, a common modality used in most hospitals, and incorporated AI technology to train the model to identify brain features such as size, shape, and texture to predict AD at a faster rate. The model was better able to identify subclinical brain shrinkage in those with early disease with 98% accuracy compared to current clinical practices that used standard hippocampal atrophy assessment (26% accuracy) and cerebrospinal beta-amyloid measurement (62% accuracy). ML algorithms have the potential to cut down diagnostic time, simplifying the diagnosis process with the use of a simple brain MRI in this instance, and greatly reduce the uncertainty of making an inaccurate diagnosis. By incorporating AI, patients could be diagnosed with AD in the early stages, allowing more time to test clinical trials of new drugs or to implement new lifestyle changes (Inglese et al., 2022). The technology demonstrates increased accuracy at diagnosing AD and would be a great service to those suffering with the disease if implemented in the clinical setting.

### Efficient analysis of data

As the number of radiographic images generated rise globally, AI has the potential to help radiologists efficiently analyze data more quickly. An IMV report on Global Imaging Department Priorities and Outlook placed improving department workflow efficiency and productivity as a top priority by radiologists (IMV Medical Information Division, 2016). Radiology departments are operating at 100% as they are dealing with COVID-19 cases and the backlog of cases that have been postponed during the pandemic. In Canada, patients were waiting an average of 50 to 82 days for CT scans and 89 days for MRI imaging prior to the COVID-19 pandemic (The Canadian Press and Germano D, 2022). Now as variants continue to overwhelm the health-care system, the problem is exacerbated with 75% of physicians in the Canadian Association of Radiologists reporting they have not reduced their backlog of medical imaging (The Canadian Press and Germano D, 2022). One way to clear the backlog is called the concept of “triaging” where the AI software decides which patients should be at the top of this list and which patients should be lowered to the bottom of the list or removed from the list (Mohan, 2018). Another study evaluated whether smart worklist prioritization by AI could optimize the radiology workflow and reduce report turnaround times (RTATs) for critical findings in chest radiographs versus the standard worklist processing called “first-in, first-out” (FIFO; Baltruschat et al., 2021). The findings showed that the average RTAT for all critical findings was significantly reduced in all prioritization simulations compared to the FIFO simulation with pneumothorax diagnostic time reduced from 80.1 min to 35.6 min. By adopting this model in the clinical setting, a worklist prioritization would allow critical patients to receive quicker diagnosis and treatment while granting more time to radiologists for other duties. With more time available, AI would allow radiologists to oversee complex imaging studies, consult with physicians, and spend more direct patient contact. In a study to determine if patients prefer to receive their results from a radiologist immediately after the examination or later from their referring physician, 92% of patients preferred to hear their results of imaging examinations from the radiologist at the time of the procedure (Schreiber et al., 1995). Therefore, with greater workflow efficiency will come greater patient satisfaction. Additionally, AI technology can improve image acquisition and quality while reducing radiation dose to patients. A group of Stanford researchers tested if using deep learning metrics with MRI and ultra-low-dose PET data could synthesize full-dose-like amyloid PET images (Chen et al., 2019). In this study, 39 patients underwent simultaneous amyloid PET/MRI examinations within a year timeframe and the data was then retrospectively analyzed. The amyloid status was tested, and the image quality was scored on a five-point scale. The PET-plus-MR images scored 3 or higher in image quality and received 89% accuracy for amyloid status compared to 91% in full-dose images. Therefore, by using deep learning methods, the researchers were able to reduce radiotracer requirements without sacrificing diagnostic quality. AI technology can also help clinicians detect hard to see lesions. At the University of Miami Miller School of Medicine, an AI tool named NeuroQuant has proven to be helpful in the assessment of dementia (McNamara, 2020). The software can help detect atrophy of certain parts of the brain in the early stages of AD that are almost impossible to recognize with the naked eye according to Dr. Saigal. The AI tool can also accurately recognize subtle changes in volume and can assess whether lesions are changing in size or number compared to prior scans. Additionally, AI technology can help identify artifacts in images and decrease false-positive interpretations. For instance, a deep learning-based tool called ArtifactID has been developed to help radiologists identify and classify artifacts in low resource settings. The researchers have trained classification models with greater than 88% accuracy to identify artifacts in T1 brain images (Manso Jimeno et al., 2022). Deep learning integration would be especially useful in developing countries where a lack of skilled physicians results in scan repetition, increased operating time, increased costs, and occasional misdiagnosis (Manso Jimeno et al., 2022). Overall, AI would greatly improve the productivity and efficiency in clinical settings.

### Reduce physician burnout

Worldwide, hospitals are performing 3.6 billion imaging procedures and producing 50 petabytes of data annually (Businesswire.com, 2021). Radiologists are tasked with collecting, reading, and processing the extensive volume of data in a timely fashion and are struggling to find efficient methods. In 2022, Medscape reported 49% of radiologists are experiencing burnout (Medscape.com, 2022). Risk factors that may contribute to physician burnout include work factors such as excessive workloads, long working hours, frequent call duties; personal characteristics associated with burnout include being self-critical, sleep deprivation, engaging in unhelpful coping strategies; and organizational factors such as negative leadership behaviors, limited interpersonal collaboration, and limited social support for physicians (Patel et al., 2018). Burnout is not only dangerous to the physician, but also to the patients. Research suggests that doctors who report signs of burnout are twice as likely to have made a medical error in the previous 3 months (Motluk, 2018). New technology could serve as a potential solution to help combat these issues and reduce burnout. AI could help radiologists by taking over tedious tasks such as scheduling patients, speeding workflows, and triaging images (Constance, 2021). Additionally, with improvement in the quality of imaging, diagnostic accuracy and efficiency will improve as well (Hardy and Harvey, 2020). Concerns about making a medical error is a major contributor to burnout and AI can help by reducing the uncertainty of a diagnosis (Robertson and Long, 2018). A new company called Rad AI is focused on empowering radiologists with AI (1231 Rad AI). Their mission is to save radiologists time, reduce burnout and help improve the quality of patient care. Rad AI Omni works by tailoring to each radiologist’s speech to automatically generate impressions and can also make follow-up recommendations for significant findings in radiology reports. Only 1 in 10 patients receive appropriate follow-up care, but with the device, adherence to follow-up recommendations is tracked to improve patient care. According to the site, Rad AI has saved over 60 min per shift, 84% of radiologists report reduced burnout, a total of 3 million impressions have been generated, and less than 220 million words have been dictated among radiologists (Rad AI, 2022). AI has the potential to relieve stress with improved efficiency and productivity. There is consensus within the medical community that in the short term, AI algorithms will serve as a tool to assist doctors rather than replace them. As AI gains popularity, radiologists are being trained to adapt to its shortcomings and strengths (Chokshi et al., 2019). The technology works optimally in collaboration with a radiologist’s extensive training and expertise as there is less productivity if either one is working alone (Langlotz, 2019). Curtis Langlotz, a radiologist at Stanford, concluded “AI will not replace radiologists, but radiologists who use AI will replace radiologists who do not”.

### Precision medicine

The “one-size-fits-all” approach in medicine is not effective in every disease. Precision, or personalized, medicine is a new form of medicine that incorporates a person’s genetic makeup, environment, and lifestyle to prevent, diagnose, and treat diseases (Ginsburg and Phillips, 2018). There have been major advances in precision medicine in the field of oncology (Mateo et al., 2022), cardiovascular disease (Abdelsayed et al., 2022) and other inflammatory diseases (Lamb et al., 2022). Alzheimer’s research is moving towards the direction of precision medicine as drug trials have failed to show success in the past decade. The aim of precision medicine in AD is to target the heterogeneity of the disease through the identification of specific pattern of risk factors and underlying pathology, and ultimately aim to form a treatment plan tailored to the individual (Reitz, 2016). Apolipoprotein E (APOE), a genetic risk factor for late-onset AD, has recently emerged as a potential target for therapeutic drug development with the use of precision medicine (Yang et al., 2021). A study using precision medicine to target key markers of AD in 25 patients was the first of its kind to reveal improvement in cognition of individuals with Alzheimer’s (Toups et al., 2021). Although a larger clinical trial is warranted, the movement towards treating AD based on a personalized approach instead of a single therapy may improve the success of drug trials in the future. AI could help precision medicine reach its full potential. Various experimental studies are attempting to converge AI and precision medicine to improve disease diagnose, risk prediction, and treatment response (Johnson et al., 2021). AI can play a major role in collecting and analyzing vast amounts of data on different individuals, help identify risk factors from clinical data, determine the effectiveness of different interventions, and provide in-depth patient characterization to develop strategies for prevention and treatment. A study used AI to create a biological sex and APOE specific network model to identify patient-specific biomarkers that could help discriminate between AD and cognitively normal individuals (Chang et al., 2023). With the assistance of AI, researchers identified key metabolic drivers that could serve as potential therapeutic targets in the future with the use of precision medicine (Chang et al., 2023). Current research raises exciting promises of integrating AI into precision medicine for personalized prevention and treatment in AD. Despite the growth in research, more studies are needed to assess the translatable potential to clinical practice and demonstrate clear evidence of real-world value.

## Challenges of AI

### Generalization and data shortage

A major limitation of AI use in the clinical setting is generalization. There are a series of steps that are implemented when creating an algorithm. The model must be trained, validated, and tested to evaluate its performance. The goal is to develop a model that is able to detect seen data and can generalize to make appropriate predictions to unseen data. The challenge with generalization arises when the trained algorithm loses its performance when applied to different datasets (Eche et al., 2021). A major cause of poor generalization is overfitting. Overfitting occurs when a model is too dependent on training dataset and is therefore not able to generalize well to data it has never seen before (Fabrizio et al., 2021). For example, a study developed a brain imaging-based classifier to assess the generalizability of machine learning for the classification of schizophrenia. The model was able to make precise predictions when using samples from a specific hospital but applying the classifier to a sample from another hospital displayed poor generalization performance (Cai et al., 2020). A possible strategy to overcome overfitting is to collect more training data (Eche et al., 2021). However, there is a lack of sufficient training data in the field of Alzheimer’s disease. The shortage of data can partly be attributed to lack of awareness or resources to refer patients to research, barriers to participation in underrepresented populations, the requirement of a study partner to report cognitive changes, participant burden, and the use of invasive procedures (Watson et al., 2014). To address this issue, data augmentation techniques can be implemented to generate new images from existing images although, the effectiveness is still unclear (Gao and Lima, 2022). Various studies have attempted to resolve this issue. A study with the largest brain MRI samples to date acquired 85,721 brain MRI samples from more than 217 scanners to build a practical AD classifier with high generalizability using deep learning/transfer leaning (Lu et al., 2022). An additional study proposed a high-generalizability machine learning framework for predicting the progression of MCI to AD using limited data (Wang et al., 2022). However, the quantity of data is not the only problem. A study evaluated the Alzheimer’s disease data landscape using nine AD cohorts including the Alzheimer’s Disease Neuroimaging Initiative (Birkenbihl et al., 2020). In addition to the lack of interoperability between the cohorts, the study revealed there is a severe overrepresentation of Caucasian individuals when compared to other ethnicities. It is predicted that by 2030, 40% of Alzheimer’s patients in the U.S. will be Latino/Black. The databases used for training are not community cohorts and are not an accurate representation of the population at large. The lack of data of non-White participants will most likely lead to poor performance in trained models when evaluating those with non-white background. There is a need for additional studies to develop algorithms with the ethnicity and demographic characteristics of subjects in mind (Bae et al., 2020). An important step is the ADNI4 project which aims to enroll 50 to 66% unrepresented populations in 2022 using new biofluid and technologies (Weiner et al., 2023). Until then, generalization will remain a challenge.

### Lack of an *in vivo* gold standard for diagnosis

Currently, there is no definitive gold standard to diagnosis AD *in vivo*. Neuropathologic evidence of extracellular amyloid plaques and intracellular neurofibrillary tangles in a post-mortem analysis remains the only gold standard (DeTure and Dickson, 2019). Scientific discoveries have identified features that are highly suggestive of AD such as amyloid, tau, and neurodegenerative changes. The National Institute on Aging and Alzheimer’s Association created a research framework for AD diagnosis using biomarker evidence to categorize individuals (Ebenau et al., 2020). The ATN (amyloid/tau/neurodegeneration) classification system includes neuroimaging and biofluids such as CSF. A study assessed the practicality of the ATN model in a longitudinal memory clinic sample and found that the A + T + N+ group accounted for the majority of patients converting to dementia (Eckerström et al., 2021). However, the ATN framework needs further validation and is not currently used in routine clinical practice. AD pathology is highly complex with evidence suggesting one can have abundant plaques without ever developing dementia (Zolochevska et al., 2018). Additionally, a study found brain changes such as small GM volumes and hypometabolic regions were associated with episodic memory loss but may be independent from Aβ pathology (Mattsson et al., 2015). Therefore, it is not enough to identify individuals at highest risk for AD with simply the presence of amyloid alone but rather, there needs to be evidence of both amyloid-β and biomarkers indicating neurodegeneration (Jagust, 2016). However, AD pathologic abnormalities have shown to differ between individuals presenting a major challenge in finding a definitive biomarker for AD diagnosis. For example, a study investigating 34 subjects with history of MCI that progressed to dementia underwent postmortem brain analysis. The study found that the majority of individuals with MCI progressed both clinically and pathologically to AD however, 29% developed non-AD primary pathologic abnormalities (Jicha et al., 2006). The study raises the question of potential pathologic heterogeneity of individuals with MCI recruited in clinical trials or AD cohort studies such as ADNI. Another study examining forty-five ADNI participants post-mortem revealed one participant only displayed non-AD pathology (argyrophilic grain disease) while comorbidities, such as Lewy body pathology, made up 58% of the cases (Franklin et al., 2015). It remains unclear whether heterogenous pathologies in AD could affect the training dataset and thus the performance of the algorithms. Without a reliable *in vivo* gold standard for diagnosis, the reliability of algorithm training is called into question.

### Skepticism in the medical community

On one end of the spectrum, many in the medical community believe AI will be highly beneficial to the field of medicine and will change the way radiologists practice - for the better. With advancing technology, medical imaging will revolutionize and improve patient outcome. However, on the other end of the spectrum, there is skepticism towards AI implementation in radiology. A literature review found four causes of skepticism towards AI from physicians: (1) concerns about the accuracy of AI in clinical settings, (2) ethical concerns such as responsibility, reliability, and transparency, (3) a sense of mistrust in the management responsible for buying and implementing AI products, and (4) fear of losing one’s skills and eventually their job (Galsgaard et al., 2022). The study suggests the loss of being in control of one’s job and a threatened self-image of being the expert when working with AI are two possible explanations behind the skepticism arising from physicians (Galsgaard et al., 2022). Worldwide, professionals in the field of radiology question whether the need for trained radiologists will be replaced by AI (Pakdemirli, 2019). Dr. Robert Schier, a neuroradiologist with RadNet Northern California, believes that in 10 to 20 years, most imaging studies will only be read by a machine and sent directly to a physician without the need of a radiologist (Wait, 2020). One study believes machine learning is the most potent threat to radiology and as the algorithm becomes more skilled in the future, it could potentially replace radiologists altogether (Chockley and Emanuel, 2016). Whether or not these concerns about AI become a reality, they must be taken seriously as there is evidence of such fears impacting medical students’ preference for radiology as their future career. A study of medical students in Saudi Arabia found that concerns of AI displacing radiologists in the future had a negative impact on choosing radiology as a specialty (Bin Dahmash et al., 2020). Of the students who chose radiology as their first specialty, 58.8% were anxious about the uncertainty of AI on radiology. Another study examined the impact of AI on US medical students’ choice of radiology as a career through an online survey distributed to 32 accredited medical schools (Reeder and Lee, 2022). The study found that AI significantly lowered students’ preference for ranking radiology with 1/6 of the students not ranking radiology as their 1^st^ choice due to AI concerns. Additionally, half of the students who considered radiology within their top three choices were concerned about AI. The study discovered that avoidance of AI within the medical student community was associated with a lower understanding of radiology, fear of decreased job opportunities, and previous AI exposure from the medical community (Reeder and Lee, 2022). The Association of American Medical Colleges predicted that by 2034, the shortage of radiologists and other specialists could surpass 35,000 (AAMC, 2021). To fill the shortage, medical students need to be persuaded into the field of radiology, but AI appears to be a deterring force. Evidently, there is skepticism towards AI implementation in radiology in the medical community. Regardless, the potential benefits of AI to patient health and outcome cannot be ignored.

### Physician bias

The use of AI in the clinical setting raises debates on risks, ethical problems, and bias. AI is expected to reduce medical errors, but this is not always the case. Potential issues that can arise are automation bias, omission errors, and commission errors. Automation bias is the tendency to favor a machine-generated diagnosis over a physician’s expertise or scientific knowledge (Neri et al., 2020). Omission errors occur when a physician rejects a correct diagnosis because he or she views AI to be without errors and does not notice or ignores its faults (Neri et al., 2020). Commission errors occur when a physician accepts the machines decision even when there are inconsistencies (Neri et al., 2020). For example, a study evaluated the influence of diagnostic suggestions on a physicians’ diagnostic decision and found that physicians are more likely to accept correct diagnoses than to reject incorrect ones (van den Berge et al., 2012). These findings indicate that suggested diagnoses may make physicians more inclined towards favoring information that confirms their belief, leading to potential diagnostic errors (van den Berge et al., 2012). The researchers also evaluated a physician’s diagnostic performance by manipulating the order in which they encountered correct or incorrect suggestions (van den Berge et al., 2012). The results indicated that the order of correct or incorrect diagnoses did not influence the physician’s tendency to accept or reject following suggestions. Overall, the study found that the physicians were more likely to accept diagnostic suggestions, regardless of whether the suggestions were correct or not (van den Berge et al., 2012). More recent studies evaluated the effect of machine-generated suggestions on physicians’ decisions. A randomized control study evaluated a physicians’ diagnostic accuracy with and without using an AI-driven differential diagnosis lists (Harada et al., 2021). The results showed that the AI driven differential diagnosis lists did not improve the physicians’ diagnostic accuracy. However, in the group with AI suggestions, the omission errors were 15.9% and the commission errors were 14.8%. The study found that commission errors tended to decrease in more experienced physicians and in those with greater mistrust in AI (Harada et al., 2021). Additionally, a study investigated the diagnostic accuracy of digital screening mammography with and without computer-aided detection (CAD; Lehman et al., 2015). The results showed that overreliance on diagnostic suggestions from a computer system resulted in more false negative rates in radiology diagnoses when compared to those without CAD (Lehman et al., 2015). Furthermore, as algorithms continue to make incorrect diagnoses, eventually people will lose trust. This is known as algorithm aversion. A study found that people are more likely to lose confidence in an algorithm than a human after seeing both make the same mistake, even if the algorithm can outperform a human in certain situations (Dietvorst et al., 2015). In conclusion, these studies raise a major problem for the use of AI in the clinical setting. If healthcare professionals become too reliant on machine diagnoses and ignore their training and the scientific evidence presented, patients’ safety will be at risk.

### Patient privacy concerns

Physicians must act by the code of medical ethics to ensure patient safety (Haskell, 2019). Patients’ right to informed consent, privacy and data protection, and ownership of healthcare information also must always be respected. One of the main limitations of AI is its requirement for large datasets to train, test, and validate the algorithms (Brady and Neri, 2020). Although the datasets have been “anonymized,” there is still significant risk for data breaches of patient privacy. An article describing the ethical issues that arise from using portable and cloud-enabled neuroimaging raises similar privacy concerns that would be expected with AI (Shen et al., 2020). The privacy concerns include questioning the effectiveness of de-identification of data, the possibility of data breach, the challenge of ensuring companies responsible for storing and analyzing patient data have adequate security, and the task of ensuring accountability to patients regarding their privacy and security (Shen et al., 2020). Additionally, while the use of such data may be beneficial to patients, there are ways to unethically make a profit on data that may cause harm to the patient. For instance, if a company chooses to purchase the rights to medical data access and capitalizes from the AI algorithm built from the data, the line is blurred regarding who should gain from the profits (Fernandez-Quilez, 2023). It may be expected that patients receive some form of payment since their data is being used. Additionally, even if the patient did not receive some compensation, the question remains whether the patient should have control or be informed of where their health information is transferred. There is no consensus regarding data ownership in these potential situations (Fernandez-Quilez, 2023). Additionally, large companies have great interest in the USD 8.3 trillion-dollar healthcare industry (Thomason, 2021). The Big Tech platforms - namely, Google, Amazon, Facebook, Apple, and Microsoft - have launched apps and medical devices to take part in the world of digital health (Thomason, 2021). Tech companies promise to protect patient healthcare records, but a survey conducted in 2018 showed Americans were least willing to share health data with tech companies at a 11% rate (Farr, 2019). The lack of trust in *big tech* is not a big surprise due to privacy scandals in the past. For instance, a healthcare company in the UK named DeepMind partnered with Royal Free London NHS Foundation Trust in 2016 to use AI in the management of acute kidney injury (Murdoch, 2021). Critics noticed that patient privacy and ownership over their health information was not adequately controlled (Murdoch, 2021). Despite the controversy, Google gained ownership over the DeepMind’s app, thereby transferring UK patient information to the US without the consent of 1.6 million patients (Murdoch, 2021). The University of College London Hospital stated they had control over the anonymized data and the information was available only to the researchers working on the project (Lomas, 2019). Yet, the term anonymized raises suspicion. While HIPPA has implemented stronger privacy protections against reidentification of data, it has loopholes and does not address the use of ML on medical data (Kancherla, 2020). For example, a 2018 study trained a ML algorithm to re-identify actual people from health tracking devices even though their data had been supposedly protected (Na et al., 2018). These findings suggest that current practices for deidentifying patient data are lacking. Until safeguards and regulations are in place, AI poses a dangerous threat to patient information, privacy, and safety.

## Future considerations

The difficultly in establishing accurate staging of AD using MRI, PET and other modalities has shifted the focus to a more precise form of imaging (Kim et al., 2022). Near-infrared fluorescence (NIRF) imaging is a powerful modality that can detect AD-associated proteins using fluorescence (Kim et al., 2022). Small molecule NIRF probes tested on AD model mice have shown success in detecting β-amyloid, tau proteins, and reactive oxygen species *in vivo* (Yang et al., 2020). A recent study developed the first NIRF probe that could image BACE1, a key protein in the pathogenic process, in live AD model mice (Bi et al., 2022). Future studies are looking to develop new NIRF probes for the diagnosis of Alzheimer’s (Quan et al., 2023). Additionally, blood tests have shown promising data for replacing more invasive PET or CSF tests and will be further investigated (Veitch et al., 2022). A recent study found blood levels of phosphorylated tau (p-tau)231 and p-tau217 could detect early cerebral Aβ pathologies demonstrating their importance as biomarkers for preclinical AD (Milà-Alomà et al., 2022). Additional future studies are needed before blood-based biomarkers are used alone to diagnose AD or incorporated in clinical care (Hansson et al., 2022). Lastly, before AI can be integrated in routine clinical setting for the diagnosis of AD, there needs to be clear evidence of real-word value. The studies presented in the paper (Table 1) illustrate various AI models that have shown high sensitivity and specificity for AD and could serve a major role in the clinical setting. However, until the models are implemented in real-world clinical trials, their validity are called into question. Further studies need to focus on improving the efficiency of AI for AD clinical trials (Seo et al., 2022). For instance, there is a need for greater external validation of the models, a need for greater quantity and quality of AD data, a need for more diversity, and a need for creating AI models based on multimodal clinical data (Acosta et al., 2022) to help bridge the translational gap. As previously mentioned, the next 5-year phase of ADNI4 will recruit more minorities and less-educated individuals to improve the generalizability of future studies (Veitch et al., 2022). Similar efforts to increase diversity are being seen in the UK, Europe, Latin America and in the Asia-Pacific region (Raman et al., 2022). Major advancements are still needed to help combat the Alzheimer’s disease crisis predicted in the coming decades.

## Conclusion

Alzheimer’s disease is a progressive, neurodegenerative disease that greatly impacts the life of the patient and the family. Signs of mental deterioration are often confused for old age and by the time the patient decides to visit the physician, a diagnosis may be too late. Neuroimaging, although costly, has served a vital role in identifying markers for the diagnosis of AD. MRI, CT, and PET are modalities used in clinical settings to identify a patient’s cognitive status, each with their own benefits and limits. As noted, limitations exist when using neuroimaging alone to identify AD. With advancing technology, there is a challenge in analyzing and interpretating vast amounts of brain imaging data. The use of AI with neuroimaging for the diagnosis of AD is a rapidly emerging field and has the potential to solve these problems. Research involving AI in neuroimaging has resulted in remarkable outcomes including the ability to classify, track the progression, and diagnose AD in early stages. These models are not currently available in routine clinical practice, but the progression towards use in clinical settings is expected as technology advances.

AI introduces limitless possibilities in the future diagnosis of AD, yet there is still resistance from the healthcare community to incorporate AI in the clinical setting. To answer the question of whether AI should be used in conjunction with neuroimaging in the diagnosis of AD, the possible benefits and challenges of AI were discussed. The main advantages of AI considered are its potential to improve diagnostic accuracy, improve the efficiency in analyzing radiographic data, reduce physician burnout, and advance precision medicine. The challenges include generalization and data shortage, lack of *in vivo* gold standard, skepticism in the medical community, potential for physician bias, and concerns over patient information, privacy, and safety. Although the pros and challenges discussed are not all specific for AD, they are still important considerations as AI will not only be used for neurocognitive diseases. It is predicted physicians of the future will be using AI in conjunction with neuroimaging in their decision-making process. However, physicians today are already incorporating AI in their workday such as with the use of triaging. Although the challenges present fundamental concerns and must be addressed when the time comes, it would be unethical not to use AI if it can improve patient health and outcome. The Hippocratic Oath taken by physicians require new physicians to uphold specific ethical standards, including to do no harm. There are risks and benefit to all medical decisions and it would ultimately be a disservice to the population if a technological advancement that could widely impact patient health for the better is not used. With anything new, resistance is an inevitable reaction as it is human nature to be uncomfortable with change. However, with change comes progress. Not only will patients greatly benefit from AI, but so too will current and future radiologists. No other field of medicine is as technologically dependent as radiology and together with AI, patient health will be greatly improved. AD is a devastating diagnosis for the patient, and for the family. No one wants to see their loved ones devoid of memories. Until there is a cure for AD, computer algorithms can play a major role in clinical settings. AI’s biggest achievements for the future of AD and other diseases are yet to be seen.

## Author contributions

SM and BA wrote the manuscript. BA supervised. All authors contributed to the article and approved the submitted version.

## Funding

CIHR (Grant number: PJT-162144)—Sex-based Differences Associated with Mitochondrial Dysfunction CAP (Grant number: 1000219580)—Effects of dietary flaxseed on memory and cognition. NSU College of Pharmacy, new lab start up funds.

## Conflict of interest

The authors declare that the research was conducted in the absence of any commercial or financial relationships that could be construed as a potential conflict of interest.

## Publisher’s note

All claims expressed in this article are solely those of the authors and do not necessarily represent those of their affiliated organizations, or those of the publisher, the editors and the reviewers. Any product that may be evaluated in this article, or claim that may be made by its manufacturer, is not guaranteed or endorsed by the publisher.

## References

[ref1] AAMC. (2021). AAMC report reinforces mounting physician shortage. Available at: https://www.aamc.org/news-insights/press-releases/aamc-report-reinforces-mounting-physician-shortage (Accessed July 22, 2022).

[ref2] Abbasi J. (2020). Neurodegenerative dementias are differentiated on PET scans. JAMA 324:2247. doi: 10.1001/jama.2020.23698 33289812

[ref3] Abdelsayed M. Kort E. J. Jovinge S. Mercola M. (2022). Repurposing drugs to treat cardiovascular disease in the era of precision medicine. Nat. Rev. Cardiol. 19, 751–764. doi: 10.1038/s41569-022-00717-6, PMID: 35606425 PMC9125554

[ref4] Acosta J. N. Falcone G. J. Rajpurkar P. Topol E. J. (2022). Multimodal biomedical AI. Nat. Med. 28, 1773–1784. doi: 10.1038/s41591-022-01981-2 36109635

[ref5] Adamo S Ramirez J Holmes MF Gao F Zotovic L Masellis M. (2020). Ventricular expansion, white matter hyperintensities, and global cognition in Alzheimer’s disease and normal aging. medRxiv 2020:2020.11.30.20240879 [Preprint]. doi: 10.1101/2020.11.30.20240879.

[ref6] Ahuja A. S. (2019). The impact of artificial intelligence in medicine on the future role of the physician. PeerJ 7:e7702. doi: 10.7717/peerj.7702, PMID: 31592346 PMC6779111

[ref7] Albert M. S. DeKosky S. T. Dickson D. Dubois B. Feldman H. H. Fox N. C.. (2011). The diagnosis of mild cognitive impairment due to Alzheimer's disease: recommendations from the National Institute on Aging-Alzheimer's Association workgroups on diagnostic guidelines for Alzheimer's disease. Alzheimers Dement. 7, 270–279. doi: 10.1016/j.jalz.2011.03.008, PMID: 21514249 PMC3312027

[ref8] Alickovic E. (2020). “For the Alzheimer’s Disease Neuroimaging Initiative, Subasi a. automatic detection of Alzheimer disease based on histogram and random forest,” in IFMBE proceedings (Switzerland: Springer International Publishing), 91–96.

[ref9] Alzheimer’s Association. (2022). Stages of Alzheimer’s. Alzheimer’s disease and dementia. Available at: https://www.alz.org/alzheimers-dementia/stages (Accessed June 19, 2022).

[ref10] Alzheimer's Association Neuroimaging Work G Albert M. (2003). The use of MRI and PET for clinical diagnosis of dementia and investigation of cognitive impairment: a consensus Report. Chicago, IL: Alzheimer's Association.

[ref11] Anand K. Sabbagh M. (2017). Amyloid imaging: poised for integration into medical practice. Neurotherapeutics 14, 54–61. doi: 10.1007/s13311-016-0474-y, PMID: 27571940 PMC5233621

[ref12] Anonymous (2018). Dementia: assessment, management and support for people living with dementia and their carers. London: National Institute for Health and Care Excellence (NICE). 30011160

[ref13] Anonymous (2022). Alzheimer’s disease facts and figures. Alzheimers Dement. 18, 700–789. doi: 10.1002/alz.12638 35289055

[ref14] Bae J. B. Lee S. Jung W. Park S. Kim W. Oh H.. (2020). Identification of Alzheimer's disease using a convolutional neural network model based on T1-weighted magnetic resonance imaging. Sci. Rep. 10:22252. doi: 10.1038/s41598-020-79243-9, PMID: 33335244 PMC7746752

[ref15] Baltruschat I. Steinmeister L. Nickisch H. Saalbach A. Grass M. Adam G.. (2021). Smart chest X-ray worklist prioritization using artificial intelligence: a clinical workflow simulation. Eur. Radiol. 31, 3837–3845. doi: 10.1007/s00330-020-07480-7, PMID: 33219850 PMC8128725

[ref16] Bao W. Xie F. Zuo C. Guan Y. Huang Y. H. (2021). PET neuroimaging of Alzheimer's disease: radiotracers and their utility in clinical research. Front. Aging Neurosci. 13:624330. doi: 10.3389/fnagi.2021.624330, PMID: 34025386 PMC8134674

[ref17] Beynon R. Sterne J. A. Wilcock G. Likeman M. Harbord R. M. Astin M.. (2012). Is MRI better than CT for detecting a vascular component to dementia? A systematic review and meta-analysis. BMC Neurol. 12:33. doi: 10.1186/1471-2377-12-33, PMID: 22672344 PMC3403932

[ref18] Bi A. Wu J. Huang S. Li Y. Zheng F. Ding J.. (2022). Functional insights from targeted imaging BACE1: the first near-infrared fluorescent probe for Alzheimer’s disease diagnosis. Biomater. Res. 26:76. doi: 10.1186/s40824-022-00320-3, PMID: 36494704 PMC9733252

[ref19] Bigler E. D. Blatter D. D. Anderson C. V. Johnson S. C. Gale S. D. Hopkins R. O.. (1997). Hippocampal volume in normal aging and traumatic brain injury. AJNR Am. J. Neuroradiol. 18, 11–23. PMID: 9010515 PMC8337859

[ref20] Bin Dahmash A. Alabdulkareem M. Alfutais A. Kamel A. M. Alkholaiwi F. Alshehri S.. (2020). Artificial intelligence in radiology: does it impact medical students preference for radiology as their future career? BJR Open 2:20200037. doi: 10.1259/bjro.20200037, PMID: 33367198 PMC7748985

[ref21] Bin Zahid A. Mikheev A. Srivatsa N. Babb J. Samadani U. Rusinek H. (2016). Accelerated brain atrophy on serial computed tomography: potential marker of the progression of Alzheimer disease. J. Comput. Assist. Tomogr. 40, 827–832. doi: 10.1097/rct.0000000000000435, PMID: 27224227 PMC5025331

[ref22] Birkenbihl C. Salimi Y. Domingo-Fernándéz D. Lovestone S. Fröhlich H. Hofmann-Apitius M.. (2020). Evaluating the Alzheimer's disease data landscape. Alzheimers Dement (N Y) 6:e12102. doi: 10.1002/trc2.12102, PMID: 33344750 PMC7744022

[ref23] Brady A. P. Neri E. (2020). Artificial intelligence in radiology—ethical considerations. Diagnostics (Basel) 10:231. doi: 10.3390/diagnostics10040231 32316503 PMC7235856

[ref24] Brisson M. Brodeur C. Létourneau-Guillon L. Masellis M. Stoessl J. Tamm A.. (2020). CCCDTD5: clinical role of neuroimaging and liquid biomarkers in patients with cognitive impairment. Alzheimers Dement (N Y) 6:e12098. doi: 10.1002/trc2.12098, PMID: 33532543 PMC7821956

[ref25] Bucci M. Chiotis K. Nordberg A. for the Alzheimer’s Disease Neuroimaging Initiative (2021). For the Alzheimer’s disease Neuroimaging I. Alzheimer’s disease profiled by fluid and imaging markers: tau PET best predicts cognitive decline. Mol. Psychiatry 26, 5888–5898. doi: 10.1038/s41380-021-01263-2, PMID: 34593971 PMC8758489

[ref26] Businesswire.com (2021). GE healthcare announces strategic collaboration agreement with AWS to transform care delivery and help clinicians improve patient care. Available at: https://www.businesswire.com/news/home/20210809005455/en/GE-Healthcare-Announces-Strategic-Collaboration-Agreement-with-AWS-to-Transform-Care-Delivery-and-Help-Clinicians-Improve-Patient-Care (Accessed July 10, 2022).

[ref27] Cai X. L. Xie D. J. Madsen K. H. Wang Y. M. Bögemann S. A. Cheung E. F. C.. (2020). Generalizability of machine learning for classification of schizophrenia based on resting-state functional MRI data. Hum. Brain Mapp. 41, 172–184. doi: 10.1002/hbm.24797, PMID: 31571320 PMC7268030

[ref28] Camicioli R. Moore M. M. Kinney A. Corbridge E. Glassberg K. Kaye J. A. (2003). Parkinson's disease is associated with hippocampal atrophy. Mov. Disord. 18, 784–790. doi: 10.1002/mds.10444 12815657

[ref29] Canary Speech (2022). Available at: https://www.canaryspeech.com (Accessed July 6, 2022).

[ref30] Carrillo M. C. Rowe C. C. Szoeke C. Masters C. L. Ames D. O'Meara T.. (2013). Research and standardization in Alzheimer’s trials: reaching international consensus. Alzheimers Dement. 9, 160–168. doi: 10.1016/j.jalz.2012.10.006, PMID: 23266004

[ref31] Castellazzi G. Cuzzoni M. G. Cotta Ramusino M. Martinelli D. Denaro F. Ricciardi A.. (2020). A machine learning approach for the differential diagnosis of Alzheimer and vascular dementia fed by MRI selected features. Front. Neuroinform. 14:25. doi: 10.3389/fninf.2020.00025, PMID: 32595465 PMC7300291

[ref32] Chandra A. Dervenoulas G. Politis M. (2019). Magnetic resonance imaging in Alzheimer's disease and mild cognitive impairment. J. Neurol. 266, 1293–1302. doi: 10.1007/s00415-018-9016-3, PMID: 30120563 PMC6517561

[ref33] Chang R. Trushina E. Zhu K. Zaidi S. S. A. Lau B. M. Kueider-Paisley A.. (2023). Predictive metabolic networks reveal sex- and APOE genotype-specific metabolic signatures and drivers for precision medicine in Alzheimer's disease. Alzheimers Dement. 19, 518–531. doi: 10.1002/alz.12675 35481667 PMC10402890

[ref34] Chen K. T. Gong E. de Carvalho Macruz F. B. Xu J. Boumis A. Khalighi M.. (2019). Ultra-low-dose (18)F-Florbetaben amyloid PET imaging using deep learning with multi-contrast MRI inputs. Radiology 290, 649–656. doi: 10.1148/radiol.2018180940, PMID: 30526350 PMC6394782

[ref35] Chiao P. Bedell B. J. Avants B. Zijdenbos A. P. Grand'Maison M. O’Neill P.. (2019). Impact of reference and target region selection on amyloid PET SUV ratios in the phase 1b PRIME study of Aducanumab. J. Nucl. Med. 60, 100–106. doi: 10.2967/jnumed.118.209130, PMID: 29777003

[ref36] Chockley K. Emanuel E. (2016). The End of Radiology? Three Threats to the Future Practice of Radiology. J Am Coll Radiol 13, 1415–1420. doi: 10.1016/j.jacr.2016.07.010 27652572

[ref37] Choi R. Y. Coyner A. S. Kalpathy-Cramer J. Chiang M. F. Campbell J. P. (2020). Introduction to machine learning, neural networks, and deep learning. Transl. Vis. Sci. Technol. 9:14. doi: 10.1167/tvst.9.2.14, PMID: 32704420 PMC7347027

[ref38] Chokshi F. H. Flanders A. E. Prevedello L. M. Langlotz C. P. (2019). Fostering a healthy AI ecosystem for radiology: conclusions of the 2018 RSNA summit on AI in radiology. Radiol Artif Intell 1:190021. doi: 10.1148/ryai.2019190021, PMID: 33937789 PMC8017423

[ref39] Computed Tomography (CT) (2022). Nih.gov. Available at: https://www.nibib.nih.gov/science-education/science-topics/computed-tomography-ct (Accessed July 1, 2022).

[ref40] Constance J.(2021). Can radiology reduce the slow burn of physician burnout? Auntminnie.com. Available at: https://www.auntminnie.com/index.aspx?sec=ser&sub=def&pag=dis&ItemID=132350 (Accessed July 10, 2022).

[ref41] Counts S. E. Ikonomovic M. D. Mercado N. Vega I. E. Mufson E. J. (2017). Biomarkers for the early detection and progression of Alzheimer's disease. Neurotherapeutics 14, 35–53. doi: 10.1007/s13311-016-0481-z, PMID: 27738903 PMC5233625

[ref42] Crous-Bou M. Minguillón C. Gramunt N. Molinuevo J. L. (2017). Alzheimer's disease prevention: from risk factors to early intervention. Alzheimers Res. Ther. 9:71. doi: 10.1186/s13195-017-0297-z, PMID: 28899416 PMC5596480

[ref43] Cuttler J. M. Lamet M. S. Calabrese E. J. (2022). Treatment of early-stage Alzheimer's disease with CT scans of the brain: a case report. Dose Response 20:15593258221078392. doi: 10.1177/15593258221078392 35321237 PMC8935565

[ref44] Damulina A. Pirpamer L. Seiler S. Benke T. Dal-Bianco P. Ransmayr G.. (2019). White matter hyperintensities in Alzheimer's disease: a lesion probability mapping study. J. Alzheimers Dis. 68, 789–796. doi: 10.3233/jad-180982, PMID: 30775995

[ref45] Dave A. Hansen N. Downey R. Johnson C. (2020). FDG-PET imaging of dementia and neurodegenerative disease. Semin. Ultrasound CT MR 41, 562–571. doi: 10.1053/j.sult.2020.08.010 33308495

[ref46] DeTure M. A. Dickson D. W. (2019). The neuropathological diagnosis of Alzheimer’s disease. Mol. Neurodegener. 14:32. doi: 10.1186/s13024-019-0333-5, PMID: 31375134 PMC6679484

[ref47] Dickerson B. C. Sperling R. A. (2008). Functional abnormalities of the medial temporal lobe memory system in mild cognitive impairment and Alzheimer's disease: insights from functional MRI studies. Neuropsychologia 46, 1624–1635. doi: 10.1016/j.neuropsychologia.2007.11.030, PMID: 18206188 PMC2760288

[ref48] Dietvorst B. J. Simmons J. P. Massey C. (2015). Algorithm aversion: people erroneously avoid algorithms after seeing them err. J. Exp. Psychol. Gen. 144, 114–126. doi: 10.1037/xge0000033, PMID: 25401381

[ref49] Ding Y. Sohn J. H. Kawczynski M. G. Trivedi H. Harnish R. Jenkins N. W.. (2019). A deep learning model to predict a diagnosis of Alzheimer disease by using (18)F-FDG PET of the brain. Radiology 290, 456–464. doi: 10.1148/radiol.2018180958, PMID: 30398430 PMC6358051

[ref50] Drzezga A. Altomare D. Festari C. Arbizu J. Orini S. Herholz K.. (2018). Diagnostic utility of 18F-Fluorodeoxyglucose positron emission tomography (FDG-PET) in asymptomatic subjects at increased risk for Alzheimer’s disease. Eur. J. Nucl. Med. Mol. Imaging 45, 1487–1496. doi: 10.1007/s00259-018-4032-1, PMID: 29756163

[ref51] Duan Y. Lin Y. Rosen D. Du J. He L. Wang Y. (2020). Identifying morphological patterns of hippocampal atrophy in patients with mesial temporal lobe epilepsy and Alzheimer disease. Front. Neurol. 11:21. doi: 10.3389/fneur.2020.00021, PMID: 32038474 PMC6989594

[ref52] Ebenau J. L. Timmers T. Wesselman L. M. P. Verberk I. M. W. Verfaillie S. C. J. Slot R. E. R.. (2020). ATN classification and clinical progression in subjective cognitive decline: the SCIENCe project. Neurology 95, e46–e58. doi: 10.1212/WNL.0000000000009724, PMID: 32522798 PMC7371376

[ref53] Eche T. Schwartz L. H. Mokrane F. Z. Dercle L. (2021). Toward generalizability in the deployment of artificial intelligence in radiology: role of computation stress testing to overcome underspecification. Radiol Artif Intell 3:e210097. doi: 10.1148/ryai.2021210097, PMID: 34870222 PMC8637230

[ref54] Eckerström C. Svensson J. Kettunen P. Jonsson M. Eckerström M. (2021). Evaluation of the ATN model in a longitudinal memory clinic sample with different underlying disorders. Alzheimers Dement (Amst) 13:e12031. doi: 10.1002/dad2.12031, PMID: 33816750 PMC8015813

[ref55] Emmady PD Schoo C Tadi P. (2022). Major neurocognitive disorder (dementia). StatPearls. Treasure Island (FL): StatPearls Publishing, StatPearls Publishing LLC. 32491376

[ref56] Evans M. C. Barnes J. Nielsen C. Kim L. G. Clegg S. L. Blair M.. (2010). Volume changes in Alzheimer's disease and mild cognitive impairment: cognitive associations. Eur. Radiol. 20, 674–682. doi: 10.1007/s00330-009-1581-5 19760240

[ref57] Eyigoz E. Mathur S. Santamaria M. Cecchi G. Naylor M. (2020). Linguistic markers predict onset of Alzheimer's disease. EClinicalMedicine 28:100583. doi: 10.1016/j.eclinm.2020.100583, PMID: 33294808 PMC7700896

[ref58] Fabrizio C. Termine A. Caltagirone C. Sancesario G. (2021). Artificial intelligence for Alzheimer's disease: promise or challenge? Diagnostics 11:1473. doi: 10.3390/diagnostics11081473, PMID: 34441407 PMC8391160

[ref59] Farr C. (2019). Tech companies see health data as a huge opportunity, but people don’t trust them. CNBC. Available at: https://www.cnbc.com/2019/02/13/consumers-dont-trust-tech-companies-with-health-data-rock-health.html (Accessed July 24, 2022).

[ref60] Fernandez-Quilez A. (2023). Deep learning in radiology: ethics of data and on the value of algorithm transparency, interpretability and explainability. AI Ethics 3, 257–265. doi: 10.1007/s43681-022-00161-9

[ref61] Ferreira L. K. Busatto G. F. (2011). Neuroimaging in Alzheimer's disease: current role in clinical practice and potential future applications. Clinics (Sao Paulo) 66, 19–24. doi: 10.1590/s1807-59322011001300003, PMID: 21779719 PMC3118433

[ref62] Ferreira D. Verhagen C. Hernández-Cabrera J. A. Cavallin L. Guo C. J. Ekman U.. (2017). Distinct subtypes of Alzheimer's disease based on patterns of brain atrophy: longitudinal trajectories and clinical applications. Sci. Rep. 7:46263. doi: 10.1038/srep46263, PMID: 28417965 PMC5394684

[ref63] Firth N. C. Primativo S. Marinescu R. V. Shakespeare T. J. Suarez-Gonzalez A. Lehmann M.. (2019). Longitudinal neuroanatomical and cognitive progression of posterior cortical atrophy. Brain 142, 2082–2095. doi: 10.1093/brain/awz136, PMID: 31219516 PMC6598737

[ref64] Ford J. N. Sweeney E. M. Skafida M. Glynn S. Amoashiy M. Lange D. J.. (2021). Heuristic scoring method utilizing FDG-PET statistical parametric mapping in the evaluation of suspected Alzheimer disease and frontotemporal lobar degeneration. Am. J. Nucl. Med. Mol. Imaging 11, 313–326. PMID: 34513285 PMC8414399

[ref65] Franklin E. E. Perrin R. J. Vincent B. Baxter M. Morris J. C. Cairns N. J.. (2015). Brain collection, standardized neuropathologic assessment, and comorbidity in Alzheimer's disease Neuroimaging initiative 2 participants. Alzheimers Dement. 11, 815–822. doi: 10.1016/j.jalz.2015.05.010, PMID: 26194314 PMC4511380

[ref66] Franzmeier N. Koutsouleris N. Benzinger T. Goate A. Karch C. M. Fagan A. M.. (2020). Predicting sporadic Alzheimer's disease progression via inherited Alzheimer's disease-informed machine-learning. Alzheimers Dement. 16, 501–511. doi: 10.1002/alz.12032, PMID: 32043733 PMC7222030

[ref67] Frisoni G. B. (2001). Structural imaging in the clinical diagnosis of Alzheimer's disease: problems and tools. J. Neurol. Neurosurg. Psychiatry 70, 711–718. doi: 10.1136/jnnp.70.6.711, PMID: 11384998 PMC1737393

[ref68] Frisoni G. B. Fox N. C. Jack C. R. Jr. Scheltens P. Thompson P. M. (2010). The clinical use of structural MRI in Alzheimer disease. Nat. Rev. Neurol. 6, 67–77. doi: 10.1038/nrneurol.2009.215, PMID: 20139996 PMC2938772

[ref69] Galsgaard A. Doorschodt T. Holten A. L. Müller F. C. Ploug Boesen M. Maas M. (2022). Artificial intelligence and multidisciplinary team meetings; a communication challenge for radiologists' sense of agency and position as spider in a web? Eur. J. Radiol. 155:110231. doi: 10.1016/j.ejrad.2022.110231 [published Online First: 20220311], PMID: 35361507

[ref70] Gao S. Lima D. (2022). A review of the application of deep learning in the detection of Alzheimer's disease. Int. J. Cogn. Comput. Eng. 3, 1–8. doi: 10.1016/j.ijcce.2021.12.002

[ref71] Garnier-Crussard A. Bougacha S. Wirth M. Dautricourt S. Sherif S. Landeau B.. (2022). White matter hyperintensity topography in Alzheimer's disease and links to cognition. Alzheimers Dement. 18, 422–433. doi: 10.1002/alz.12410, PMID: 34322985 PMC9292254

[ref72] Ginsburg G. S. Phillips K. A. (2018). Precision medicine: from science to value. Health Aff (Millwood) 37, 694–701. doi: 10.1377/hlthaff.2017.1624, PMID: 29733705 PMC5989714

[ref73] Golla S. S. Verfaillie S. C. Boellaard R.. (2019). Quantification of [(18)F]florbetapir: a test-retest tracer kinetic modelling study. J. Cereb. Blood Flow Metab. 39, 2172–2180. doi: 10.1177/0271678X18783628, PMID: 29897009 PMC6826855

[ref74] Gunes S. Aizawa Y. Sugashi T. Sugimoto M. Rodrigues P. P. (2022). Biomarkers for Alzheimer's disease in the current state: a narrative review. Int. J. Mol. Sci. 23. doi: 10.3390/ijms23094962, PMID: 35563350 PMC9102515

[ref75] Guzmán-Vélez E. Diez I. Schoemaker D. Pardilla-Delgado E. Vila-Castelar C. Fox-Fuller J. T.. (2022). Amyloid-β and tau pathologies relate to distinctive brain dysconnectomics in preclinical autosomal-dominant Alzheimer’s disease. Proc. Natl. Acad. Sci. U. S. A. 119:e2113641119. doi: 10.1073/pnas.2113641119, PMID: 35380901 PMC9169643

[ref76] Hanseeuw B. J. Betensky R. A. Jacobs H. I. L. Schultz A. P. Sepulcre J. Becker J. A.. (2019). Association of Amyloid and tau with Cognition in preclinical Alzheimer disease: a longitudinal study. JAMA Neurol. 76, 915–924. doi: 10.1001/jamaneurol.2019.1424, PMID: 31157827 PMC6547132

[ref77] Hansson O. Edelmayer R. M. Boxer A. L. Carrillo M. C. Mielke M. M. Rabinovici G. D.. (2022). The Alzheimer's Association appropriate use recommendations for blood biomarkers in Alzheimer's disease. Alzheimers Dement. 18, 2669–2686. doi: 10.1002/alz.12756, PMID: 35908251 PMC10087669

[ref78] Harada Y. Katsukura S. Kawamura R. Shimizu T. (2021). Efficacy of artificial-intelligence-driven differential-diagnosis list on the diagnostic accuracy of physicians: an open-label randomized controlled study. Int. J. Environ. Res. Public Health 18. doi: 10.3390/ijerph18042086, PMID: 33669930 PMC7924871

[ref79] Hardy M. Harvey H. (2020). Artificial intelligence in diagnostic imaging: impact on the radiography profession. Br. J. Radiol. 93:20190840. doi: 10.1259/bjr.20190840, PMID: 31821024 PMC7362930

[ref80] Haskell S. L. (2019). Medical ethics in radiography. Radiol. Technol. 90, 237–254. PMID: 30635456

[ref81] Hausman H. K. Hardcastle C. Kraft J. N. Evangelista N. D. Boutzoukas E. M. O'Shea A.. (2022). The association between head motion during functional magnetic resonance imaging and executive functioning in older adults. Neuroimage Rep. 2:100085. doi: 10.1016/j.ynirp.2022.100085 PMC10299743 37377763

[ref82] Health Quality Ontario (2014). The appropriate use of neuroimaging in the diagnostic work-up of dementia: an evidence-based analysis. Ont Health Technol Assess Ser 14, 1–64. PMC3937983 24592296

[ref83] Herholz K. Salmon E. Perani D. Baron J. C. Holthoff V. Frölich L.. (2002). Discrimination between Alzheimer dementia and controls by automated analysis of multicenter FDG PET. NeuroImage 17, 302–316. doi: 10.1006/nimg.2002.1208, PMID: 12482085

[ref84] Herring W. (2011). Learning radiology: Recognizing the basics E-book (2nd ed.). Philadelphia, PA: Saunders.

[ref85] IMV Medical Information Division. (2016). Market reports on medical imaging & clinical diagnostic markets. Available at: https://imvinfo.com (Accessed July 10, 2022).

[ref86] Inglese M. Patel N. Linton-Reid K. Loreto F. Win Z. Perry R. J.. (2022). A predictive model using the mesoscopic architecture of the living brain to detect Alzheimer's disease. Commun Med (Lond) 2:70. doi: 10.1038/s43856-022-00133-4, PMID: 35759330 PMC9209493

[ref87] Jagust W. (2016). Is amyloid-β harmful to the brain? Brain Commun 139, 23–30. doi: 10.1093/brain/awv326, PMID: 26614753 PMC4990654

[ref88] James C. Ranson J. M. Everson R. Llewellyn D. J. (2021). Performance of machine learning algorithms for predicting progression to dementia in memory clinic patients. JAMA Netw. Open 4:e2136553. doi: 10.1001/jamanetworkopen.2021.36553, PMID: 34913981 PMC8678688

[ref89] Jebelli J. Hamper M. C. Van Quelef D. Caraballo D. Hartmann J. Kumi-Diaka J. (2022). The potential therapeutic effects of low-dose ionizing radiation in Alzheimer's disease. Cureus 14:e23461. doi: 10.7759/cureus.23461, PMID: 35371871 PMC8958987

[ref90] Jicha G. A. Parisi J. E. Dickson D. W. Johnson K. Cha R. Ivnik R. J.. (2006). Neuropathologic outcome of mild cognitive impairment following progression to clinical dementia. Arch. Neurol. 63, 674–681. doi: 10.1001/archneur.63.5.674, PMID: 16682537

[ref91] Jie C. Treyer V. Schibli R. Mu L. (2021). Tauvid™: The first FDA-approved PET tracer for imaging tau pathology in Alzheimer's disease. Pharmaceuticals (Basel) 14. doi: 10.3390/ph14020110, PMID: 33573211 PMC7911942

[ref92] Johnson K. A. Davis K. R. Buonanno F. S. Brady T. J. Rosen T. J. Growdon J. H. (1987). Comparison of magnetic resonance and roentgen ray computed tomography in dementia. Arch. Neurol. 44, 1075–1080. doi: 10.1001/archneur.1987.00520220071020, PMID: 3632382

[ref93] Johnson K. A. Fox N. C. Sperling R. A. Klunk W. E. (2012). Brain imaging in Alzheimer disease. Cold Spring Harb. Perspect. Med. 2:a006213. doi: 10.1101/cshperspect.a006213, PMID: 22474610 PMC3312396

[ref94] Johnson K. B. Wei W. Q. Weeraratne D. Frisse M. E. Misulis K. Rhee K.. (2021). Precision medicine, AI, and the future of personalized health care. Clin. Transl. Sci. 14, 86–93. doi: 10.1111/cts.12884, PMID: 32961010 PMC7877825

[ref95] Kancherla J. (2020). Re-identification of health data through machine learning. Social Science Electronic Publishing presents Social Science Research Network. doi: 10.2139/ssrn.3794927

[ref96] Kapadnis M. N. Bhattacharyya A. Subasi A. (2023). “Artificial intelligence based Alzheimer’s disease detection using deep feature extraction” in Applications of artificial intelligence in medical imaging (London, United Kingdom: Academic Press Elsevier), 333–355.

[ref97] Kapoor M Kasi A. (2022). PET Scanning. StatPearls. Treasure Island (FL): StatPearls Publishing, StatPearls Publishing LLC..

[ref98] Kavitha C. Mani V. Srividhya S. R. Khalaf O. I. Tavera Romero C. A. (2022). Early-stage Alzheimer's disease prediction using machine learning models. Front. Public Health 10:853294. doi: 10.3389/fpubh.2022.853294 [published Online First: 20220303] 35309200 PMC8927715

[ref99] Khosravi M. Peter J. Wintering N. A. Serruya M. Shamchi S. P. Werner T. J.. (2019). 18F-FDG is a superior indicator of cognitive performance compared to 18F-Florbetapir in Alzheimer’s disease and mild cognitive impairment evaluation: a global quantitative analysis. J. Alzheimers Dis. 70, 1197–1207. doi: 10.3233/JAD-190220, PMID: 31322568

[ref100] Kim J. Jeong M. Stiles W. R. Choi H. S. (2022). Neuroimaging modalities in Alzheimer's disease: diagnosis and clinical features. Int. J. Mol. Sci. 23:6079. doi: 10.3390/ijms23116079, PMID: 35682758 PMC9181385

[ref101] Kim J. P. Kim J. Park Y. H. Park S. B. Lee J. S. Yoo S.. (2019). Machine learning based hierarchical classification of frontotemporal dementia and Alzheimer's disease. Neuroimage Clin. 23:101811. doi: 10.1016/j.nicl.2019.101811, PMID: 30981204 PMC6458431

[ref102] Kolanko M. A. Win Z. Loreto F. Patel N. Carswell C. Gontsarova A.. (2020). Amyloid PET imaging in clinical practice. Pract. Neurol. 20, 451–462. doi: 10.1136/practneurol-2019-002468 32973035

[ref103] Koolschijn P. C. van Haren N. E. Cahn W. Schnack H. G. Janssen J. Klumpers F.. (2010). Hippocampal volume change in schizophrenia. J. Clin. Psychiatry 71, 737–744. doi: 10.4088/JCP.08m04574yel 20492835

[ref104] Koscik R. L. Betthauser T. J. Jonaitis E. M. Allison S. L. Clark L. R. Hermann B. P.. (2020). Amyloid duration is associated with preclinical cognitive decline and tau PET. Alzheimers Dement (Amst) 12:e12007. doi: 10.1002/dad2.12007, PMID: 32211502 PMC7085284

[ref105] Krishnadas N. Villemagne V. L. Doré V. Rowe C. C. (2021). Advances in brain amyloid imaging. Semin. Nucl. Med. 51, 241–252. doi: 10.1053/j.semnuclmed.2020.12.005 33482999

[ref106] Lamb C. A. Saifuddin A. Powell N. Rieder F. (2022). The future of precision medicine to predict outcomes and control tissue remodeling in inflammatory bowel disease. Gastroenterology 162, 1525–1542. doi: 10.1053/j.gastro.2021.09.077, PMID: 34995532 PMC8983496

[ref107] Langlotz C. P. (2019). Will artificial intelligence replace radiologists? Radiol Artif Intell 1:e190058. doi: 10.1148/ryai.2019190058, PMID: 33937794 PMC8017417

[ref108] Lehman C. D. Wellman R. D. Buist D. S. Kerlikowske K. Tosteson A. N. Miglioretti D. L.. (2015). Diagnostic accuracy of digital screening mammography with and without computer-aided detection. JAMA Intern. Med. 175, 1828–1837. doi: 10.1001/jamainternmed.2015.5231, PMID: 26414882 PMC4836172

[ref109] Lerch J. P. Pruessner J. C. Zijdenbos A. Hampel H. Teipel S. J. Evans A. C. (2005). Focal decline of cortical thickness in Alzheimer's disease identified by computational neuroanatomy. Cereb. Cortex 15, 995–1001. doi: 10.1093/cercor/bhh200, PMID: 15537673

[ref110] Levin F. Ferreira D. Lange C. Dyrba M. Westman E. Buchert R.. (2021). Data-driven FDG-PET subtypes of Alzheimer’s disease-related neurodegeneration. Alzheimers Res. Ther. 13:49. doi: 10.1186/s13195-021-00785-9, PMID: 33608059 PMC7896407

[ref111] Lomas N. (2019). Researchers spotlight the lie of ‘anonymous’ data. TechCrunch. Available at: https://social.techcrunch.com/2019/07/24/researchers-spotlight-the-lie-of-anonymous-data/ (Accessed July 24, 2022).

[ref112] Lu B. Li H.-X. Chang Z.-K. Li L. Chen N. X. Zhu Z. C.. (2022). A practical Alzheimer’s disease classifier via brain imaging-based deep learning on 85,721 samples. J. Big Data 9:101. doi: 10.1186/s40537-022-00650-y

[ref113] Lu D. Popuri K. Ding G. W. Balachandar R. Beg M. F. Alzheimer’s Disease Neuroimaging Initiative. (2018). Multimodal and multiscale deep neural networks for the early diagnosis of Alzheimer's disease using structural MR and FDG-PET images. Sci. Rep. 8:5697. doi: 10.1038/s41598-018-22871-z, PMID: 29632364 PMC5890270

[ref114] Luxenberg J. S. Haxby J. V. Creasey H. Sundaram M. Rapoport S. I. (1987). Rate of ventricular enlargement in dementia of the Alzheimer type correlates with rate of neuropsychological deterioration. Neurology 37, 1135–1140. doi: 10.1212/wnl.37.7.1135, PMID: 3496557

[ref115] Magnetic Resonance Imaging (MRI) (2022). Nih.gov. Available at: https://www.nibib.nih.gov/science-education/science-topics/magnetic-resonance-imaging-mri (Accessed June 30, 2022).

[ref116] Manso Jimeno M. Ravi K. S. Jin Z. Oyekunle D. Ogbole G. Geethanath S. (2022). ArtifactID: identifying artifacts in low-field MRI of the brain using deep learning. Magn. Reson. Imaging 89, 42–48. doi: 10.1016/j.mri.2022.02.002, PMID: 35176447

[ref117] Márquez F. Yassa M. A. (2019). Neuroimaging biomarkers for Alzheimer’s disease. Mol. Neurodegener. 14:21. doi: 10.1186/s13024-019-0325-5, PMID: 31174557 PMC6555939

[ref118] Mateo J. Steuten L. Aftimos P. André F. Davies M. Garralda E.. (2022). Delivering precision oncology to patients with cancer. Nat. Med. 28, 658–665. doi: 10.1038/s41591-022-01717-2 35440717

[ref119] Mattsson N. Insel P. S. Aisen P. S. Jagust W. Mackin S. Weiner M.. (2015). Brain structure and function as mediators of the effects of amyloid on memory. Neurology 84, 1136–1144. doi: 10.1212/WNL.0000000000001375 25681451 PMC4371407

[ref120] McKhann G. M. Knopman D. S. Chertkow H. Hyman B. T. Jack C. R. Jr. Kawas C. H.. (2011). The diagnosis of dementia due to Alzheimer's disease: recommendations from the National Institute on Aging-Alzheimer's Association workgroups on diagnostic guidelines for Alzheimer's disease. Alzheimers Dement. 7, 263–269. doi: 10.1016/j.jalz.2011.03.005, PMID: 21514250 PMC3312024

[ref121] McNamara D. (2020). New AI technology can help assess brain lesions and atrophy. InventUM | University of Miami Miller School of medicine. Available at: https://physician-news.umiamihealth.org/new-ai-technology-can-help-assess-brain-lesions-and-atrophy/ (Accessed July 10, 2022).

[ref122] Medscape.com (2022). Medscape radiologist lifestyle, happiness and burnout report of 2022. Available at: https://www.medscape.com/slideshow/2022-lifestyle-radiologist-6014784 (Accessed July 10, 2022).

[ref123] Mielke M. M. Hagen C. E. Wennberg A. M. V.. (2017). Association of plasma Total tau level with cognitive decline and risk of mild cognitive impairment or dementia in the Mayo Clinic study on aging. JAMA Neurol. 74, 1073–1080. doi: 10.1001/jamaneurol.2017.1359 28692710 PMC5710182

[ref124] Milà-Alomà M. Ashton N. J. Shekari M. Salvadó G. Ortiz-Romero P. Montoliu-Gaya L.. (2022). Plasma p-tau231 and p-tau217 as state markers of amyloid-β pathology in preclinical Alzheimer’s disease. Nat. Med. 28, 1797–1801. doi: 10.1038/s41591-022-01925-w, PMID: 35953717 PMC9499867

[ref125] Minoshima S. Cross D. Thientunyakit T. Foster N. L. Drzezga A. (2022). F-FDG PET imaging in neurodegenerative dementing disorders: insights into subtype classification, emerging disease categories, and mixed dementia with copathologies. J. Nucl. Med. 63:2S. doi: 10.2967/jnumed.121.263194 35649653

[ref126] Minoshima S. Giordani B. Berent S. Frey K. A. Foster N. L. Kuhl D. E. (1997). Metabolic reduction in the posterior cingulate cortex in very early Alzheimer's disease. Ann. Neurol. 42, 85–94. doi: 10.1002/ana.410420114 9225689

[ref127] Mohan C. S. (2018). Artificial intelligence in radiology—are we treating the image or the patient? Indian J Radiol Imaging 28, 137–139. doi: 10.4103/ijri.IJRI_256_18, PMID: 30050233 PMC6038229

[ref128] Moscoso A. Rey-Bretal D. Silva-Rodríguez J. Aldrey J. M. Cortés J. Pías-Peleteiro J.. (2020). White matter hyperintensities are associated with subthreshold amyloid accumulation. NeuroImage 218:116944. doi: 10.1016/j.neuroimage.2020.116944, PMID: 32445880

[ref129] Moscoso A. Silva-Rodríguez J. Aldrey J. M. Cortés J. Pías-Peleteiro J. M. Ruibal Á.. (2022). 18F-florbetapir PET as a marker of myelin integrity across the Alzheimer’s disease spectrum. Eur. J. Nucl. Med. Mol. Imaging 49, 1242–1253. doi: 10.1007/s00259-021-05493-y, PMID: 34581847 PMC8921113

[ref130] Motluk A. (2018). Do doctors experiencing burnout make more errors? CMAJ 190, E1216–E1217. doi: 10.1503/cmaj.109-5663, PMID: 30301750 PMC6175626

[ref131] Murdoch B. (2021). Privacy and artificial intelligence: challenges for protecting health information in a new era. BMC Med. Ethics 22:122. doi: 10.1186/s12910-021-00687-3, PMID: 34525993 PMC8442400

[ref132] Na L. Yang C. Lo C.-C. Zhao F. Fukuoka Y. Aswani A. (2018). Feasibility of Reidentifying individuals in large National Physical Activity Data Sets from Which Protected Health Information has Been Removed with use of machine learning. JAMA Netw. Open 1:e186040. doi: 10.1001/jamanetworkopen.2018.6040, PMID: 30646312 PMC6324329

[ref133] Neri E. Coppola F. Miele V. Bibbolino C. Grassi R. (2020). Artificial intelligence: who is responsible for the diagnosis? Radiol. Med. 125, 517–521. doi: 10.1007/s11547-020-01135-9 32006241

[ref134] Nestor S. M. Rupsingh R. Borrie M. Smith M. Accomazzi V. Wells J. L.. (2008). Ventricular enlargement as a possible measure of Alzheimer's disease progression validated using the Alzheimer's disease neuroimaging initiative database. Brain 131, 2443–2454. doi: 10.1093/brain/awn146, PMID: 18669512 PMC2724905

[ref135] Nugent S. Croteau E. Potvin O. Castellano C. A. Dieumegarde L. Cunnane S. C.. (2020). Selection of the optimal intensity normalization region for FDG-PET studies of normal aging and Alzheimer’s disease. Sci. Rep. 10:9261. doi: 10.1038/s41598-020-65957-3, PMID: 32518360 PMC7283334

[ref136] Odusami M. Maskeliūnas R. Damaševičius R. Krilavičius T. (2021). Analysis of features of Alzheimer's disease: detection of early stage from functional brain changes in magnetic resonance images using a Finetuned ResNet18 network. Diagnostics (Basel) 11. doi: 10.3390/diagnostics11061071, PMID: 34200832 PMC8230447

[ref137] Okudzhava L. Heldmann M. Münte T. F. (2022). A systematic review of diffusion tensor imaging studies in obesity. Obes. Rev. 23:e13388. doi: 10.1111/obr.13388, PMID: 34908217

[ref138] Oosterman J. M. Oosterveld S. Rikkert M. G. Claassen J. A. Kessels R. P. (2012). Medial temporal lobe atrophy relates to executive dysfunction in Alzheimer's disease. Int. Psychogeriatr. 24, 1474–1482. doi: 10.1017/s1041610212000506, PMID: 22717328

[ref139] Orringer D. A. Vago D. R. Golby A. J. (2013). Clinical applications and future directions of functional MRI. Semin. Neurol. 32, 466–475. doi: 10.1055/s-0032-1331816 PMC3787513 23361489

[ref140] Ossenkoppele R. Pichet Binette A. Groot C. Smith R. Strandberg O. Palmqvist S.. (2022). Amyloid and tau PET-positive cognitively unimpaired individuals are at high risk for future cognitive decline. Nat. Med. 28, 2381–2387. doi: 10.1038/s41591-022-02049-x, PMID: 36357681 PMC9671808

[ref141] Pakdemirli E. (2019). Artificial intelligence in radiology: friend or foe? Where are we now and where are we heading? Acta Radiol Open 8:205846011983022. doi: 10.1177/2058460119830222 PMC6385326 30815280

[ref142] Pasi M. Poggesi A. Pantoni L. (2011). The use of CT in dementia. Int. Psychogeriatr. 23, S6–S12. doi: 10.1017/s1041610211000950 21729420

[ref143] Patel R. S. Bachu R. Adikey A. Malik M. Shah M. (2018). Factors related to physician burnout and its consequences: a review. Behav Sci (Basel) 8. doi: 10.3390/bs8110098, PMID: 30366419 PMC6262585

[ref144] Pearce M. S. Salotti J. A. Little M. P. McHugh K. Lee C. Kim K. P.. (2012). Radiation exposure from CT scans in childhood and subsequent risk of leukaemia and brain tumours: a retrospective cohort study. Lancet 380, 499–505. doi: 10.1016/s0140-6736(12)60815-0, PMID: 22681860 PMC3418594

[ref145] Pietroboni A. M. Colombi A. Carandini T. Sacchi L. Fenoglio C. Marotta G.. (2022). Amyloid PET imaging and dementias: potential applications in detecting and quantifying early white matter damage. Alzheimers Res. Ther. 14:33. doi: 10.1186/s13195-021-00933-1, PMID: 35151361 PMC8841045

[ref146] Quan L. Moreno-Gonzalez I. Xie Z. Gamez N. Vegas-Gomez L. Song Q.. (2023). A near-infrared probe for detecting and interposing amyloid beta oligomerization in early Alzheimer's disease. Alzheimers Dement. 19, 456–466. doi: 10.1002/alz.12673 35436382

[ref147] Rad AI. (2022). Radai.com. Available at: https://www.radai.com (Accessed July 10, 2022).

[ref148] Raman R. Aisen P. S. Carillo M. C. Detke M. Grill J. D. Okonkwo O. C.. (2022). Tackling a major deficiency of diversity in Alzheimer's disease therapeutic trials: an CTAD task force report. J. Prev Alzheimers Dis. 9, 388–392. doi: 10.14283/jpad.2022.50, PMID: 35841239 PMC9098373

[ref149] Ranzenberger LR Snyder T. (2022). Diffusion tensor imaging. StatPearls. Treasure Island (FL): StatPearls publishing, StatPearls Publishing LLC. 30726046

[ref150] Reeder K. Lee H. (2022). Impact of artificial intelligence on US medical students' choice of radiology. Clin. Imaging 81, 67–71. doi: 10.1016/j.clinimag.2021.09.018, PMID: 34619566

[ref151] Reitz C. (2016). Toward precision medicine in Alzheimer's disease. Ann Transl Med 4:107. doi: 10.21037/atm.2016.03.05, PMID: 27127760 PMC4828743

[ref152] Rentz D. M. Papp K. V. Mayblyum D. V. Sanchez J. S. Klein H. Souillard-Mandar W.. (2021). Association of Digital Clock Drawing with PET amyloid and tau pathology in Normal older adults. Neurology 96, e1844–e1854. doi: 10.1212/WNL.0000000000011697, PMID: 33589537 PMC8105970

[ref153] Robertson J. J. Long B. (2018). Suffering in silence: medical error and its impact on health care providers. J. Emerg. Med. 54, 402–409. doi: 10.1016/j.jemermed.2017.12.001, PMID: 29366616

[ref154] Sammet S. (2016). Magnetic resonance safety. Abdom Radiol (NY) 41, 444–451. doi: 10.1007/s00261-016-0680-4, PMID: 26940331 PMC4848040

[ref155] Sampath R. Indumathi J. (2018). Earlier detection of Alzheimer disease using N-fold cross validation approach. J. Med. Syst. 42:217. doi: 10.1007/s10916-018-1068-5, PMID: 30280260

[ref156] Schreiber M. H. Leonard M. Jr. Rieniets C. Y. (1995). Disclosure of imaging findings to patients directly by radiologists: survey of patients' preferences. AJR Am. J. Roentgenol. 165, 467–469. doi: 10.2214/ajr.165.2.7618577, PMID: 7618577

[ref157] Schwarz C. G. (2021). Uses of human MR and PET imaging in research of neurodegenerative brain diseases. Neurotherapeutics 18, 661–672. doi: 10.1007/s13311-021-01030-9, PMID: 33723751 PMC8423895

[ref158] Seo Y. Jang H. Lee H. (2022). Potential applications of artificial intelligence in clinical trials for Alzheimer's disease. Life (Basel) 12:275. doi: 10.3390/life12020275, PMID: 35207561 PMC8879055

[ref159] Sexton C. E. Kalu U. G. Filippini N. Mackay C. E. Ebmeier K. P. (2011). A meta-analysis of diffusion tensor imaging in mild cognitive impairment and Alzheimer's disease. Neurobiol. Aging 32, 2322.e5–2322.e18. doi: 10.1016/j.neurobiolaging.2010.05.019, PMID: 20619504

[ref160] Shamim S. Hasler G. Liew C. Sato S. Theodore W. H. (2009). Temporal lobe epilepsy, depression, and hippocampal volume. Epilepsia 50, 1067–1071. doi: 10.1111/j.1528-1167.2008.01883.x, PMID: 19054394 PMC2692336

[ref161] Sheikh Z. Glick Y. (2022). Cerebral atrophy. Available at: Radiopaedia.org (Accessed July 1, 2022).

[ref162] Sheikh-Bahaei N. Sajjadi S. A. Manavaki R. Gillard J. H. (2017). Imaging biomarkers in Alzheimer’s disease: a practical guide for clinicians. J. Alzheimer's Dis. Rep. 1, 71–88. doi: 10.3233/ADR-170013, PMID: 30480230 PMC6159632

[ref163] Shen F. X. Wolf S. M. Gonzalez R. G. Garwood M. (2020). Ethical issues posed by field research using highly portable and cloud-enabled Neuroimaging. Neuron 105, 771–775. doi: 10.1016/j.neuron.2020.01.041, PMID: 32135089 PMC8803403

[ref164] Shivamurthy V. K. N. Tahari A. K. Marcus C. Subramaniam R. M. (2015). Brain FDG PET and the diagnosis of dementia. Am. J. Roentgenol. 204, W76–W85. doi: 10.2214/AJR.13.12363 25539279

[ref165] Silva-Spínola A. Baldeiras I. Arrais J. P. Santana I. (2022). The road to personalized medicine in Alzheimer’s disease: the use of artificial intelligence. Biomedicine 10:315. doi: 10.3390/biomedicines10020315, PMID: 35203524 PMC8869403

[ref166] Smailagic N. Lafortune L. Kelly S. Hyde C. Brayne C. (2018). 18F-FDG PET for prediction of conversion to Alzheimer's disease dementia in people with mild cognitive impairment: an updated systematic review of test accuracy. J. Alzheimers Dis. 64, 1175–1194. doi: 10.3233/jad-171125, PMID: 30010119 PMC6218118

[ref167] Smith N. M. Ford J. N. Haghdel A. Glodzik L. Li Y. D’Angelo D.. (2022). Statistical parametric mapping in amyloid positron emission tomography. Front. Aging Neurosci. 14:849932. doi: 10.3389/fnagi.2022.849932, PMID: 35547630 PMC9083453

[ref168] Specht K. (2020). Current challenges in translational and clinical fMRI and future directions. Front. Psych. 10:924. doi: 10.3389/fpsyt.2019.00924 PMC6960120 31969840

[ref169] Subasi A. (2020). “Chapter 11—use of artificial intelligence in Alzheimer’s disease detection” in Artificial intelligence in precision health. ed. Barh D. (Cambridge, MA: Academic Press), 257–278.

[ref170] Subasi A. Kapadnis M. N. Kosal B. A. (2022). “4—Alzheimer’s disease detection using artificial intelligence” in Augmenting neurological disorder prediction and rehabilitation using artificial intelligence. eds. Pillai A. S. Menon B. (Cambridge, MA: Academic Press), 53–74.

[ref171] Terry D. P. Sabatinelli D. Puente A. N. Lazar N. A. Miller L. S. (2015). A meta-analysis of fMRI activation differences during episodic memory in Alzheimer's disease and mild cognitive impairment. J. Neuroimaging 25, 849–860. doi: 10.1111/jon.12266, PMID: 26076800

[ref172] Mirabnahrazam G. Ma D. Lee S. Popuri K. Lee H. Cao J.. (2022). Machine learning based multimodal Neuroimaging genomics dementia score for predicting future conversion to Alzheimer's disease. J. Alzheimers Dis. 87, 1345–1365. doi: 10.3233/jad-220021, PMID: 35466939 PMC9195128

[ref173] The Canadian Press and Germano D (2022). Radiologists warn of growing backlog in medical imaging due to COVID-19 pandemic. CTV News. Available at: https://www.ctvnews.ca/health/radiologists-warn-of-growing-backlog-in-medical-imaging-due-to-covid-19-pandemic-1.5736702 (Accessed July 10, 2022).

[ref174] Thomason J. (2021). Big tech, big data and the new world of digital health. Global Health J. 5, 165–168. doi: 10.1016/j.glohj.2021.11.003

[ref175] Toups K Hathaway A Gordon D. (2021). Precision medicine approach to Alzheimer’s disease: successful proof-of-concept trial. medRxiv 2021:2021.05.10.21256982. doi: 10.1101/2021.05.10.21256982.

[ref176] Turk K. W. Vives-Rodriguez A. Schiloski K. A. Marin A. Wang R. Singh P.. (2022). Amyloid PET ordering practices in a memory disorders clinic. Alzheimers Dement (N Y) 8:e12333. doi: 10.1002/trc2.12333, PMID: 35992217 PMC9382692

[ref177] van den Berge K. Mamede S. van Gog T. Romijn J. A. van Guldener C. van Saase J. L. C. M.. (2012). Accepting diagnostic suggestions by residents: a potential cause of diagnostic error in medicine. Teach. Learn. Med. 24, 149–154. doi: 10.1080/10401334.2012.664970, PMID: 22490096

[ref178] van den Berge K. Mamede S. van Gog T. van Saase J. Rikers R. (2012). Consistency in diagnostic suggestions does not influence the tendency to accept them. Can Med Educ J 3, e98–e106. doi: 10.36834/cmej.36594 26451191 PMC4563636

[ref179] van Oostveen W. M. de Lange E. C. M. (2021). Imaging techniques in Alzheimer's disease: a review of applications in early diagnosis and longitudinal monitoring. Int. J. Mol. Sci. 22. doi: 10.3390/ijms22042110, PMID: 33672696 PMC7924338

[ref180] Veitch D. P. Weiner M. W. Aisen P. S. Beckett L. A. DeCarli C. Green R. C.. (2022). Using the Alzheimer's disease Neuroimaging initiative to improve early detection, diagnosis, and treatment of Alzheimer's disease. Alzheimers Dement. 18, 824–857. doi: 10.1002/alz.12422, PMID: 34581485 PMC9158456

[ref181] Wait. (2020). Will AI replace radiologists after all? Radiology Business. Available at: https://radiologybusiness.com/topics/artificial-intelligence/wait-will-ai-replace-radiologists-after-all (Accessed July 22, 2022).

[ref182] Wang C. Li Y. Tsuboshita Y. Sakurai T. Goto T. Yamaguchi H.. (2022). A high-generalizability machine learning framework for predicting the progression of Alzheimer's disease using limited data. NPJ Digit Med 5:43. doi: 10.1038/s41746-022-00577-x, PMID: 35414651 PMC9005545

[ref183] Watson J. L. Ryan L. Silverberg N. Cahan V. Bernard M. A. (2014). Obstacles and opportunities in Alzheimer's clinical trial recruitment. Health Aff (Millwood) 33, 574–579. doi: 10.1377/hlthaff.2013.1314, PMID: 24711317 PMC4167360

[ref184] Weigand A. J. Maass A. Eglit G. L. Bondi M. W. (2022). What’s the cut-point? A systematic investigation of tau PET thresholding methods. Alzheimers Res. Ther. 14:49. doi: 10.1186/s13195-022-00986-w, PMID: 35382866 PMC8985353

[ref185] Weiner M. W. Veitch D. P. Miller M. J. Aisen P. S. Albala B. Beckett L. A.. (2023). Increasing participant diversity in AD research: plans for digital screening, blood testing, and a community-engaged approach in the Alzheimer's disease Neuroimaging initiative 4. Alzheimers Dement. 19, 307–317. doi: 10.1002/alz.12797, PMID: 36209495 PMC10042173

[ref186] Yang A. Kantor B. Chiba-Falek O. (2021). APOE: the new frontier in the development of a therapeutic target towards precision medicine in late-onset Alzheimer's. Int. J. Mol. Sci. 22. doi: 10.3390/ijms22031244, PMID: 33513969 PMC7865856

[ref187] Yang J. Zeng F. Ge Y. Peng K. Li X. Li Y.. (2020). Development of near-infrared fluorescent probes for use in Alzheimer’s disease diagnosis. Bioconjug. Chem. 31, 2–15. doi: 10.1021/acs.bioconjchem.9b00695, PMID: 31769660

[ref188] Yousefzadeh-Nowshahr E. Winter G. Bohn P. Kneer K. von Arnim C. A. F. Otto M.. (2022). Quantitative analysis of regional distribution of tau pathology with 11C-PBB3-PET in a clinical setting. PLoS One 17:e0266906. doi: 10.1371/journal.pone.0266906, PMID: 35404966 PMC9045369

[ref189] Zaharchuk G. Gong E. Wintermark M. Rubin D. Langlotz C. P. (2018). Deep learning in neuroradiology. AJNR Am. J. Neuroradiol. 39, 1776–1784. doi: 10.3174/ajnr.A5543, PMID: 29419402 PMC7410723

[ref190] Zhang J. Zheng B. Gao A. Feng X. Liang D. Long X. (2021). A 3D densely connected convolution neural network with connection-wise attention mechanism for Alzheimer's disease classification. Magn. Reson. Imaging 78, 119–126. doi: 10.1016/j.mri.2021.02.001, PMID: 33588019

[ref191] Zhu Q. Wang Y. Zhuo C. Xu Q. Yao Y. Liu Z.. (2022). Classification of Alzheimer's disease based on abnormal hippocampal functional connectivity and machine learning. Front. Aging Neurosci. 14:754334. doi: 10.3389/fnagi.2022.754334, PMID: 35273489 PMC8902140

[ref192] Zolochevska O. Bjorklund N. Woltjer R. Wiktorowicz J. E. Taglialatela G. (2018). Postsynaptic proteome of non-demented individuals with Alzheimer's disease neuropathology. J. Alzheimers Dis. 65, 659–682. doi: 10.3233/jad-180179, PMID: 30103319 PMC6130411

